# Genome-wide characterization and expression profiling of *PDI* family gene reveals function as abiotic and biotic stress tolerance in Chinese cabbage (*Brassica rapa* ssp*. pekinensis*)

**DOI:** 10.1186/s12864-017-4277-2

**Published:** 2017-11-16

**Authors:** Md. Abdul Kayum, Jong-In Park, Ujjal Kumar Nath, Gopal Saha, Manosh Kumar Biswas, Hoy-Taek Kim, Ill-Sup Nou

**Affiliations:** 1 Department of Horticulture, Sunchon National University, 255 Jungang-ro, Suncheon, Jeonnam 57922 Republic of Korea; 2 University-Industry Cooperation Foundation, Sunchon National University, 255 Jungang-ro, Suncheon, Jeonnam 57922 Republic of Korea

**Keywords:** Pdi, Chinese cabbage, Gene expression, Gene evolution, Biotic and abiotic stresses

## Abstract

**Background:**

Protein disulfide isomerase (PDI) and PDI-like proteins contain thioredoxin domains that catalyze protein disulfide bond, inhibit aggregation of misfolded proteins, and function in isomerization during protein folding in endoplasmic reticulum and responses during abiotic stresses.Chinese cabbage is widely recognized as an economically important, nutritious vegetable, but its yield is severely hampered by various biotic and abiotic stresses. Because of, it is prime need to identify those genes whose are responsible for biotic and abiotic stress tolerance. PDI family genes are among of them.

**Results:**

We have identified 32 *PDI* genes from the Br135K microarray dataset, NCBI and BRAD database, and in silico characterized their sequences. Expression profiling of those genes was performed using cDNA of plant samples imposed to abiotic stresses; cold, salt, drought and ABA (Abscisic Acid) and biotic stress; *Fusarium oxysporum* f. sp. *conglutinans* infection. The Chinese cabbage *PDI* genes were clustered in eleven groups in phylogeny. Among them, 15 *PDI* genes were ubiquitously expressed in various organs, while 24 *PDI* genes were up-regulated under salt and drought stress. By contrast, cold and ABA stress responsive gene number were ten and nine, respectively. In case of *F. oxysporum* f. sp. *conglutinans* infection 14 *BrPDI* genes were highly up-regulated. Interestingly, *BrPDI1–1* gene was identified as putative candidate against abiotic (salt and drought) and biotic stresses, *BrPDI5–2* gene for ABA stress, and *BrPDI1–4, 6–1* and *9–2* were putative candidate genes for both cold and chilling injury stresses.

**Conclusions:**

Our findings help to elucidate the involvement of *PDI* genes in stress responses, and they lay the foundation for functional genomics in future studies and molecular breeding of *Brassica rapa* crops. The stress-responsive *PDI* genes could be potential resources for molecular breeding of *Brassica* crops resistant to biotic and abiotic stresses.

**Electronic supplementary material:**

The online version of this article (10.1186/s12864-017-4277-2) contains supplementary material, which is available to authorized users.

## Background

Protein disulfide isomerases (PDIs) are enzymes found primarily in the endoplasmic reticulum (ER) in eukaryotes play a vital role in protein folding. Proteins, immediately after biosynthesis must be folded into correct three-dimensional shape for proper functioning. Misfolded proteins are often nonfunctional by producing aggregates and interferes cellular functions. PDIs can bind into misfolded or unfolded proteins preventing to produce such aggregates [1]. Venetianer and Straub, [2] firstly identified PDIs as protein-folding catalysts and demonstrated their catastrophic consequences of a defective protein folding process. Plants utilize multiple mechanisms to fold proteins correctly for proper functioning. PDIs are one of the mechanisms, which work through formation and breakage of connections between cysteine residues by producing disulfide bonds, which was described by Anfinsen [3]. These bonds stabilize the folded protein and provide correct structure to perform its particular function.

PDIs contain four thioredoxin (TRX)-like domains, two of which contain a catalytic site for disulfide bond formation. The reduced (dithiol) forms of PDIs catalyze the reduction of impaired thiol residues of a particular substrate, acting as an isomerase [4]. In human, PDIs usually contain four TRX-like domains (a, b, b’ and a’), a linker (x) and a C-terminal extension domain (c) [5]. The ‘a’ and ‘a’ domains are TRX domains, containing an active Cys-Gly-His-Cys motif joined to α-helices and β-strands (β-α-β-α-β-α-β-β-α) that is essential for polypeptide redox and isomerization [6]. Although the ‘*b*’ and ‘*b’* domains share some structural identity with TRX domain, they do not possess specific active motif site [6]. The α-helices are particularly important for DNA binding motifs, including helix-turn-helix motifs, leucine zipper motifs and zinc finger motifs. The helix-turn-helix (HTH) motif is a major structural motif capable of DNA binding. This motif is composed of two α-helices joined by a short strand of amino acids found in many regulatory proteins for gene expression [7]. The C-terminal region contains a C-domain rich in acidic residues typical as calcium binding proteins and ends with an ER retention signal composed of four amino acids, generally KDEL/GKNF/VASS [8].

*PDI* genes have been identified in many higher plants, such as 21 *PDI* genes in *Arabidopsis thaliana*, 12 in *Oryza sativa*, 12 in *Zea mays,* 10 in *Vitis vinifera*, 9 in *Triticum aestivum* and so on [9]. In eukaryotes, PDI family proteins are divided into eleven groups based on phylogenetic analysis. Proteins in groups’ I–V contain two thioredoxin-like active domains, whereas those in groups VI–XI contain a single thioredoxin-like active domain. PDI proteins in higher plants are involved in signal transduction pathways and in transcriptional complexes that regulate the responses of genes to environmental stimuli. PDI proteins ERP57, PDIp, P5, ERP72, PDIR, and PDI-D act as redox catalysts and isomerases and exhibit differential functions, such as peptide binding, cell adhesion, and chaperone activities [10]. These proteins play key role as storage protein and plasma membrane maturation [9] *GmPDIL-3a* and *GmPDIL-3b* are highly expressed during seed maturation suggesting that they are involved in folding or accumulation of storage proteins in soybean [11]. *PDIL2–1* of *A. thaliana* is directly involved in ovule structure and embryo sac development and determining proper direction of pollen tube growth [12]. The PDI-like protein RB60 plays important roles providing redox potential to regulate photosynthesis [13]. Most wheat *PDI* and *PDIL* genes are expressed during endosperm development, indicating their association with storage protein biosynthesis and deposition, which is directly related to gluten quality [14]. *BdPDIL1–1* is significantly up-regulated under abiotic (drought, salt, ABA and H_2_O_2_) stress, suggesting their involvement in multiple stress responses [15]. All maize *PDI* genes are highly responsive to cold, salt, dehydration, and ABA stress [16]. During drought, heat and cold stress, *TaPDI1*, *TaPDI2* and *TaPDI3* are highly up-regulated in roots and other tissues [17]. There are many family genes involved in tolerance to abiotic and biotic stresses in plants; however we have selected PDI because of its abundance of ubiquitous sulfydryl oxidoreductase protein, which is an important cellular protein with multiple biological functions of all eukaryotic cells. This sulfydryl oxidoreductase protein of PDI displays versatile redox behavior, which also interact with other proteins and assumed its potential role against various diseases and abiotic stresses [18]. In addition, PDI is an important redox proteins regulating reactive oxygen species (ROS) production in the cells and alter redox status of cells to activate defense mechanism [19]. In this study, we make effort to identify the members of *PDI* gene family in *Brassica rapa* through genome wide exploration by using different bioinformatic tools. In addition, the putative candidates of *PDI* genes responsive to abiotic and biotic stresses are predicted through expression profiling using stress induced Chinese cabbage materials.

## Results

### Identification of *PDI* genes in *B. rapa*

A total of 32 *BrPDI* genes were identified using SWISSPROT of the *B. rapa* genomic database (http://brassicadb.org/brad/searchAll.php) (BRAD; [20]); using key word “PDI”, NCBI, and annotations of microarray Br135K dataset from cold-treated *B. rapa* (Chiifu & Kenshin), removing any duplicates. A BLAST search was performed using *Arabidopsis* PDI sequences as the query, and picked the encoded protein and genomic sequences of *PDI* genes from BRAD database for 32 *BrPDI* genes. A high degree of similarity of these 32 BrPDI protein sequences was also picked for other plant species. Isoelectric points, molecular weights and residual size (ranged from 144 to 596 aa) of putative 32 BrPDI proteins are presented in Table 1.

**Table 1:** In silico analysis of PDI genes identified in Chinese cabbage with their *Arabidopsis* orthologs

Gene name	Gene ID	Chromosome			Stand	Sub-genome	Iso electric point (Pi)	Molecular weight (Mw)	Protein length	Orthologous gene
		No.	Start	End						
BrPDI1–1	Bra016405	A08	17,525,813	17,528,427	–	MF1	4.66	55.78	501	AT1G21750
BrPDI1–2	Bra012293	A07	11,001,072	11,003,432	–	MF2	4.92	55.04	496	AT1G21750
BrPDI1–3	Bra017948	A06	8,725,538	8,728,086	+	LF	5.21	55.52	498	AT1G21750
BrPDI1–4	Bra008311	A02	13,630,848	13,633,652	+	MF1	4.79	56.24	511	AT1G77510
BrPDI1–5	Bra015665	A07	24,746,925	24,749,554	+	LF	4.85	55.8	509	AT1G77510
BrPDI2–1	Bra007120	A09	28,945,749	28,948,773	–	LF	4.70	64.28	579	AT3G54960
BrPDI2–2	Bra002464	A10	9,445,124	9,447,998	–	LF	4.59	66.16	596	AT5G60640
BrPDI2–3	Bra020239	A02	4,767,927	4,771,274	+	MF2	4.58	65.06	588	AT5G60640
BrPDI3–1	Bra014319	A08	1,670,650	1,673,502	–	MF1	4.74	46.36	413	AT1G52260
BrPDI3–2	Bra018958	A06	1,024,377	1,027,518	–	LF	4.98	59.04	529	AT1G52260
BrPDI4–1	Bra000454	A03	11,214,808	11,217,122	+	MF2	5.80	39.48	362	AT2G47470
BrPDI4–2	Bra004455	A05	202,365	204,674	–	LF	6.05	39.45	361	AT2G47470
BrPDI5–1	Bra015375	A10	1,739,233	1,741,963	+	LF	6.43	46.64	432	AT1G04980
BrPDI5–2	Bra005546	A05	6,147,811	6,150,674	+	LF	5.71	47.85	443	AT2G32920
BrPDI6–1	Bra018672	A06	2,704,568	2,705,424	–	LF	4.93	16.43	144	AT1G07960
BrPDI7–1	Bra034408	A05	13,683,334	13,685,270	–	MF2	5.28	49.08	435	AT1G35620
BrPDI8–1	Bra001793	A03	18,521,034	18,525,290	+	MF2	6.85	54.29	484	AT3G20560
BrPDI8–2	Bra035770	A05	17,795,565	17,799,462	–	LF	6.96	54.31	483	AT3G20560
BrPDI8–3	Bra019071	A03	26,497,077	26,500,750	+	MF1	6.82	53.75	478	AT4G27080
BrPDI8–4	Bra010413	A08	13,621,192	13,624,765	–	MF2	6.72	53.89	480	AT4G27080
BrPDI8–5	Bra030465	A05	11,654,854	11,657,768	–	MF2	7.73	54.20	483	AT1G50950
BrPDI8–6	Bra018881	A06	1,468,257	1,472,162	–	LF	6.26	53.57	477	AT1G50950
BrPDI9–1	Bra026786	A09	35,442,828	35,445,717	+	MF2	6.85	60.45	532	AT1G15020
BrPDI9–2	Bra014330	A08	1,583,063	1,585,667	+	MF1	6.65	58.34	523	AT2G01270
BrPDI10–1	Bra001092	A03	14,721,770	14,723,230	+	MF2	6.75	33.86	303	AT3G03860
BrPDI10–2	Bra031969	A05	24,598,071	24,599,646	–	MF1	7.09	34.22	302	AT3G03860
BrPDI10–3	Bra036758	A08	7,293,480	7,294,725	+	MF1	8.57	33.54	298	AT3G03860
BrPDI10–4	Bra036429	A01	26,156,526	26,158,281	_	LF	8.91	36.75	326	AT1G34780
BrPDI11–1	Bra019406	A03	24,393,210	24,394,793	_	MF1	6.02	51.69	468	AT4G21990
BrPDI11–2	Bra013579	A01	6,445,389	6,446,955	_	LF	6.39	51.58	468	AT4G21990
BrPDI11–3	Bra034466	Scaffold000096	53,632	55,924	_	LF	6.74	53.67	479	AT1G62180
BrPDI11–4	Bra029505	A09	17,850,361	17,852,000	+	LF	6.74	53.57	479	AT4G04610

### Phylogenetic analysis and domain location

**Fig. 1:**
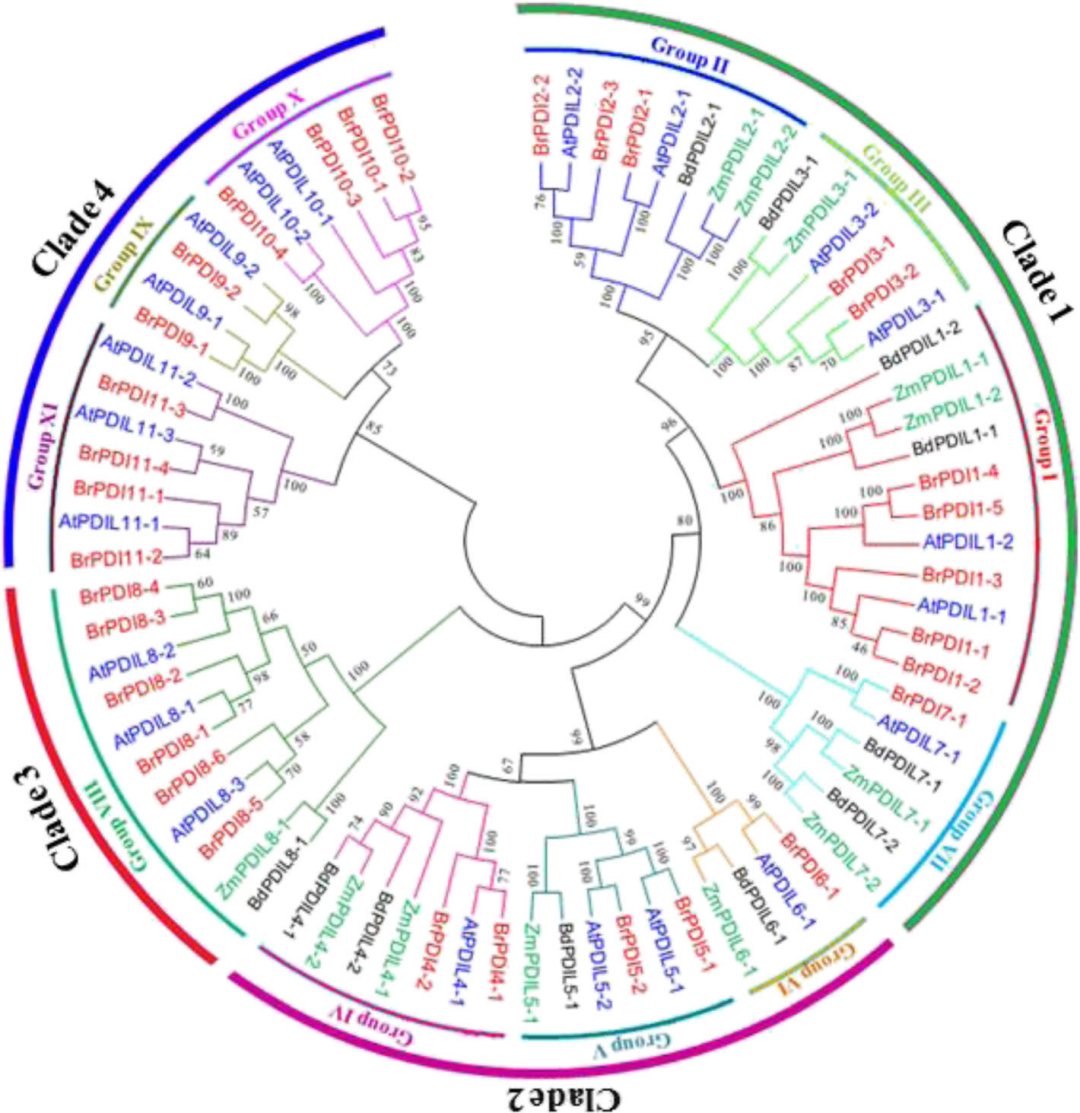
Phylogenetic analysis of PDI protein identified in *B. rapa, Arabidopsis, Brachypodium distachyon* and *Zea mays*. The phylogenetic tree was generated by the neighbor-joining method in MEGA6.0 with 1000 bootstrap replicates using full-length PDI protein sequences from *B. rapa, Arabidopsis, B. distachyon* and *Z. mays.* Blue*,* red, Green, and black letters indicate *Arabidopsis, B. rapa, Z. mays* and *B. distachyon* proteins, respectively

**Fig. 2:**
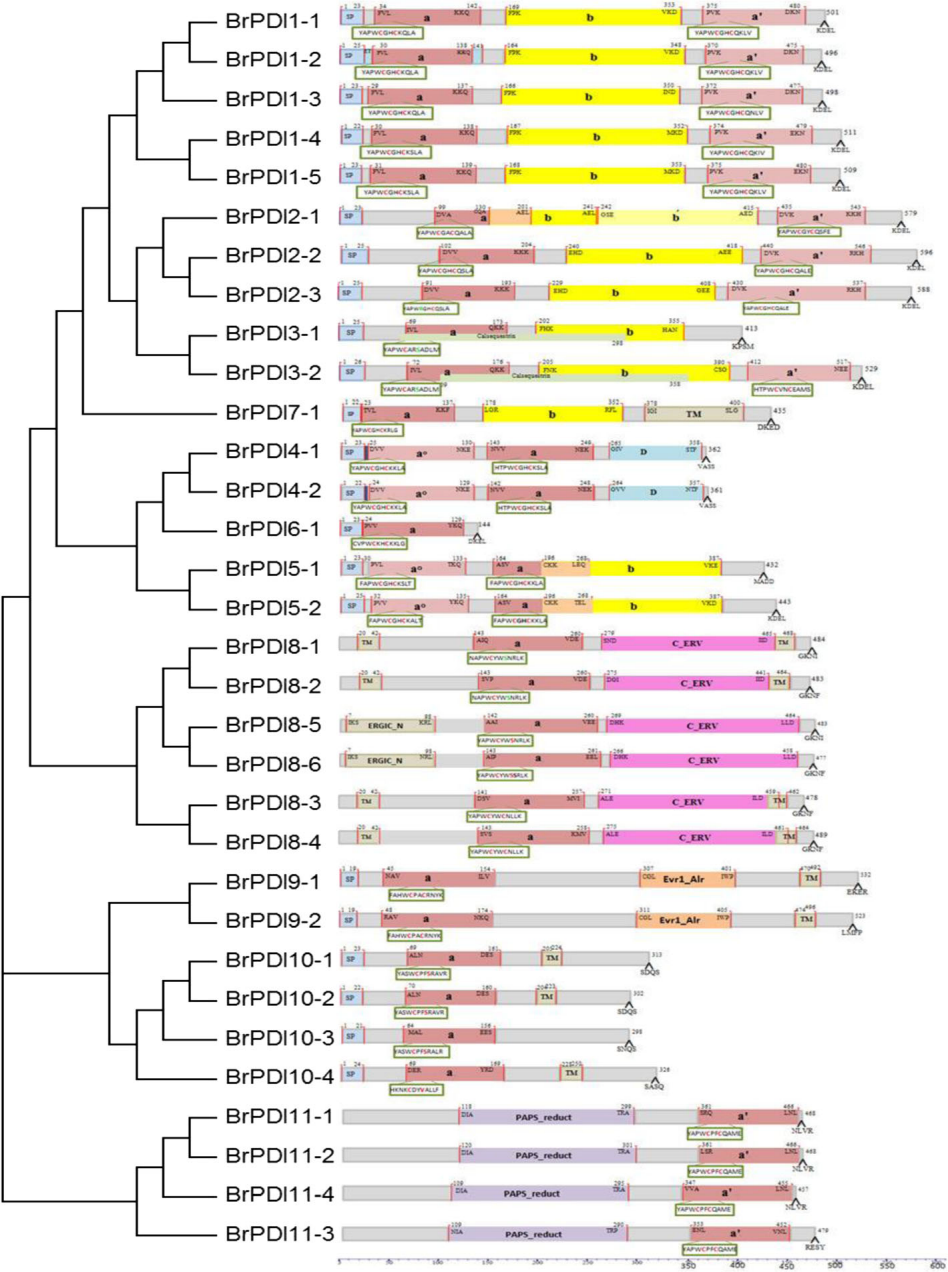
Domain structure of the deduced amino acid sequences of *B. rapa PDI* genes. The putative signal peptides (SP), the a and b type domains, the N-terminal calcium binding domain calsequestrin, the D domains (Erp29), the transmembrane domains (TM) and the C_ERV (COPII-coatedERV) domain are shown. The thioredoxin-like catalytic domains with two active sites (shown in detail in the boxes) are also shown. Numbers above indicate domain boundaries (aa), and numbers on the right indicate ORF (aa). Domains a^o^ (light gray) and a’ (gray) are homologous to TRX and contain the catalytic CxxC motif (red). Domains b (yellow) and b’ (light yellow) also exhibit a TRX fold, but they do not share high sequence similarity with each other or with domains a or a’. The C-terminal extension (red) contains a (K/H)DEL retention signal for the ER

**Fig. 3:**
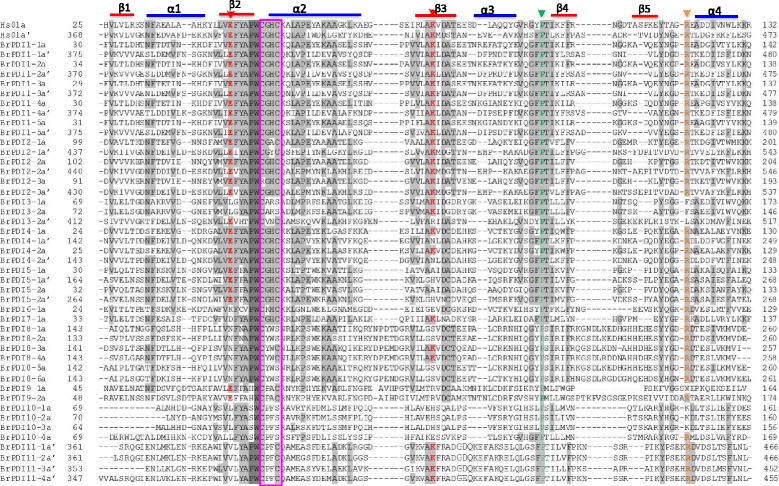
Multiple sequence alignment of the a-type domains of *B. rapa* PDI proteins and a typical human PDI. Residues highlighted in gray and light gray share 100% and >50% identity, respectively. Elements of the secondary structure are specified by blue colour bars (α-helices) and red colour bars (β-sheets). Red arrows indicate the two buried charged residues in the vicinity of the active site, orange arrow indicates the conserved arginine (R) and green arrow indicates the *cis* prolines (P) near each active site. Active-site residues within a domain are pink colour boxed

A phylogenetic tree was constructed using 76 full-length protein sequences of *PDI* and *PDI-like* genes, which included 32 from Chinese cabbage, 11 from *Brachypodium distachyon,* 12 from maize, and 21 from *Arabidopsis* to investigate evolutionary relationship. Eleven phylogenetic groups were denoted among the considered *PDI* genes from different plant species (Fig. 1) with distinct 4 clades. Clade 1 consisted phylogenetic groups I, II, III and VII, whose members contain two active thioredoxin domains, whereas members of group VII encoded proteins with a single N-terminal active domain (Fig. 2). Clade 2 contained phylogenetic group IV, V and VI, among them members of group IV and V possessing two active thioredoxin domains in tandem at their N-termini. Group members of VI may have lost one of the two active thioredoxin domains due to shared structural features of a common progenitor. Clade 3 contained phylogenetic groups VIII, which included genes encoding proteins with a single active thioredoxin domain. The members of groups IX, X and XI formed clade 4; encoded proteins with an active thioredoxin domain. Phylogenetic and domain analyses revealed that these *PDI* family genes are divergent in plant. Most BrPDI proteins are contained an N-terminal signal peptide and a C-terminal KDEL signal responsible for translocation and ER retention, respectively. In the domain structures, ‘a’ and ‘a’ are homologous to thioredoxin and contain a -CXXC- active site for isomerase and redox activities. By contrast, the ‘b’ and ‘b’ domains have no homology to thioredoxin and lack of -CXXC- catalytic site. However, the secondary structures of four (a, a’, b, b’) domains are similar to thioredoxin rather than the active catalytic site. The thioredoxin-like domain comprises *βαβαβαββα* motifs forming four layers of *β*-sheets that are sandwiched three layers of *α*-helixes (Fig. 3).

We performed multiple sequence alignment of ‘a’- and ‘a’-type domain sequences of BrPDI proteins to analyze the features of them. These domain sequences contained five β-sheets, four α-helices and the -CGHC- motif of the active site. They also contained conserved arginine, glutamic acid, proline, and lysine residues (Fig. 3). All BrPDI members ‘a’-type domain with conserved arginine residue, critical for their catalytic function, except BrPDI3–1 and BrPDI3–2 which contained phenylalanine or leucine residues instead of arginine residue. In addition, BrPDI9–1 contained tryptophan and BrPDI10–1, BrPDI10–2 and BrPDI10–3 contained lysine in the place of arginine residues. BrPDI2–3 accomplished with RGHC non-characteristic active sites; but BrPDI3–1 and BrPDI3–2 with CARS. Whereas, BrPDI8–1, BrPDI8–2, BrPDI8–5, and BrPDI8–6 comprised with CYWS; and BrPDI10–1, BrPDI10–2, BrPDI10–3, and BrPDI10–4 contained CPFS non-characteristic active sites (Fig. 2) which may affect their redox potential and lose their function.

### Chromosomal distribution of *BrPDI* genes

**Fig. 4:**
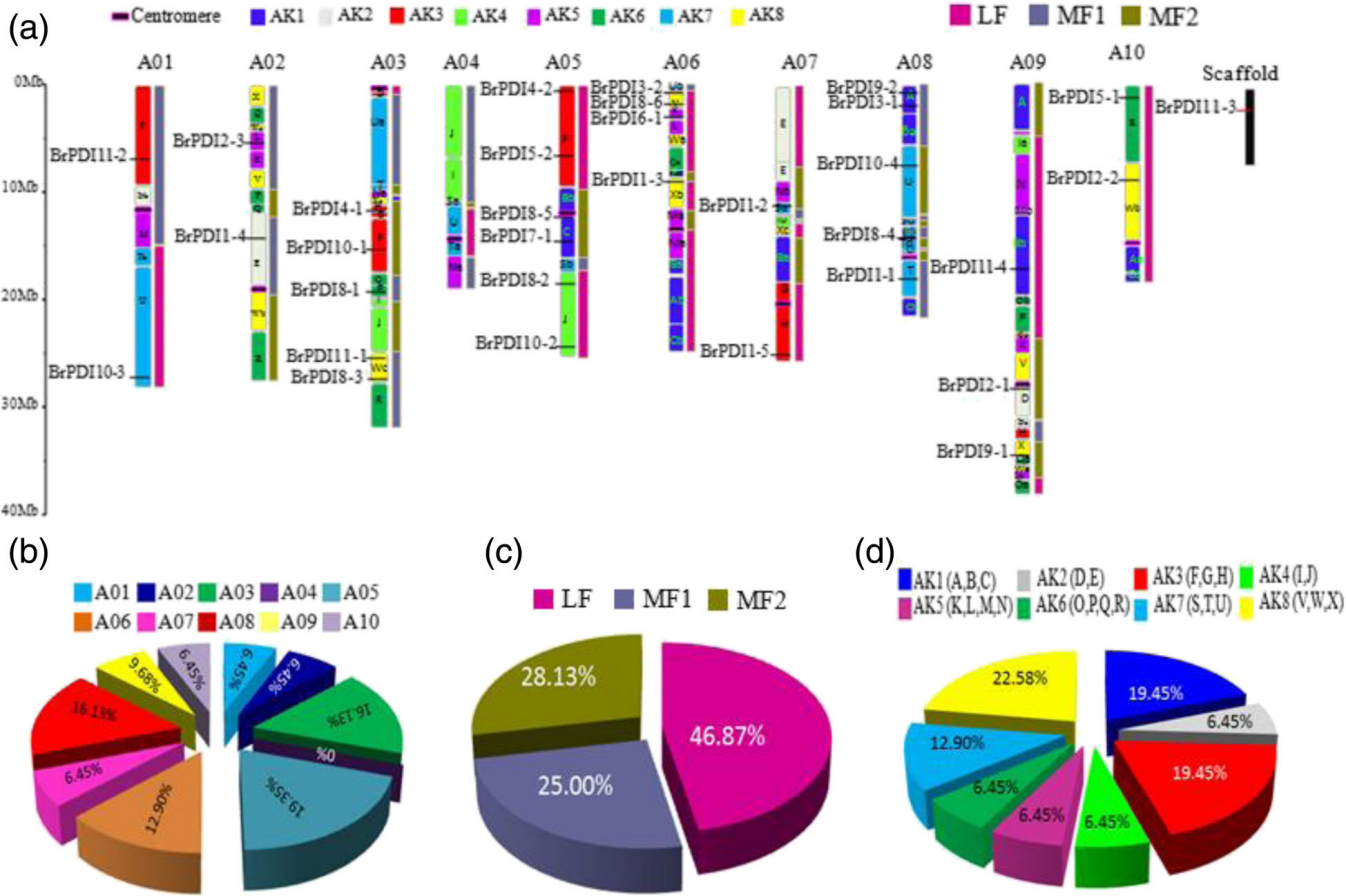
**a** Distribution of *BrPDI* genes on 10 chromosomes. The 24 (A to X) ancestral blocks and three sub-genomes are plotted, based on the report of Schranz et al., [46]. **b** The percentages of *BrPDI* genes on ancestral blocks are demonstrated in graph. **c** The percentages of *BrPDI* genes on each subgenomes are demonstrated in pie graph. Least fractionated (LF), medium fractionated (MF1) and most fractionated (MF2) genomes. **d** The percentages of *BrPDI* genes on each chromosome are also demonstrated in pie graph

Thirty two *BrPDI* genes are located on different chromosomes not less than one on each except chromosome 04. The highest number (six) of *BrPDI* genes was identified on chromosome A05 (19.35%), while chromosome A01, A02, A07, and A10 contained two *BrPDI* genes (6.45%) for each, and chromosome A04 has no *BrPDI* genes (0%). Only one gene (*BrPDI11–3*) is located in scaffold (Fig. 4a and b). None of the *BrPDI* gene clusters was detected in Chinese cabbage. The sequence alignment of BrPDI proteins showed higher similarity within groups. BrPDI proteins showed ≥68% similarity within groups, except group10 and group 2. By contrast, similarity between groups was ≤65%. Seventeen pairs of BrPDI proteins showed ˃80% similarity indicating duplications are predominant for those genes (Table 2).

**Table 2:** For each pair wise alignment, the similarity (relative to the maximum similarity) and the number of identical amino acids (in %) is given

Protein	BrPDI 1–2	BrPDI 1–3	BrPDI 1–4	BrPDI 1–5	BrPDI 2–1	BrPDI 2–2	BrPDI 2–3	BrPDI 3–1	BrPDI 3–2	BrPDI 4–1	BrPDI 4–2	BrPDI 5–1	BrPDI 5–2	BrPDI 6–1	BrPDI 7–1	BrPDI 8–1	BrPDI 8–2	BrPDI 8–3	BrPDI 8–4	BrPDI 8–5	BrPDI 8–6	BrPDI9–1	BrPDI 9–2	BrPDI 10–1	BrPDI 10–2	BrPDI 10–3	BrPDI 10–4	BrPDI 11–1	BrPDI 11–2	BrPDI 11–3	BrPDI 11–4
BrPDI1–1	**90**	**84**	73	76	30	33	32	19	21	24	26	19	18	25	20	9	9	12	10	32	32	26	18	20	23	24	28	27	25	28	30
BrPDI1–2		**83**	74	77	32	33	32	18	18	25	26	20	19	25	20	8	12	12	10	32	32	25	19	20	24	23	29	29	29	32	34
BrPDI1–3			72	74	28	31	32	19	19	24	26	19	19	24	21	9	9	10	10	27	32	26	22	21	24	24	29	27	25	27	29
BrPDI1–4				**82**	28	32	33	19	17	28	26	21	20	21	16	12	12	12	11	33	31	28	20	20	20	24	27	23	23	24	25
BrPDI1–5					29	29	31	17	21	24	25	21	19	25	19	11	12	12	13	34	32	29	20	20	24	24	27	31	25	24	27
BrPDI2–1						55	54	28	29	25	25	18	21	27	19	11	15	11	14	27	28	29	23	22	23	22	24	36	35	38	36
BrPDI2–2							**86**	30	30	27	28	20	18	22	17	11	13	12	13	33	31	25	23	23	20	21	26	34	33	37	41
BrPDI2–3								29	28	27	28	19	18	27	20	12	11	14	14	31	30	24	22	23	17	20	25	37	32	37	34
BrPDI3–1									**80**	18	14	15	15	19	16	13	11	11	11	26	25	25	21	25	26	29	25	35	32	34	30
BrPDI3–2										18	17	16	16	23	18	9	8	11	9	26	26	24	12	29	29	31	29	31	33	40	30
BrPDI4–1											**93**	28	23	31	18	13	13	14	15	31	35	27	15	27	26	27	31	29	29	28	29
BrPDI4–2												29	25	34	15	13	13	16	16	33	37	32	14	27	25	27	31	30	28	28	28
BrPDI5–1													**81**	27	15	11	13	15	13	29	32	29	16	25	26	27	27	34	33	32	29
BrPDI5–2														23	13	13	14	13	10	28	27	27	14	27	28	29	25	30	30	34	30
BrPDI6–1															26	27	27	28	29	31	34	27	21	27	32	22	23	25	25	29	32
BrPDI7–1																12	11	11	11	21	23	23	16	26	19	20	25	29	27	29	26
BrPDI8–1																	**86**	70	70	74	69	31	18	28	25	23	23	25	28	23	30
BrPDI8–2																		68	69	74	70	31	16	27	23	27	41	28	24	24	26
BrPDI8–3																			**89**	68	65	32	11	65	22	22	41	24	23	23	27
BrPDI8–4																				67	63	32	18	65	23	25	41	26	26	26	25
BrPDI8–5																					**82**	31	14	24	25	26	47	23	27	23	26
BrPDI8–6																						35	16	25	28	25	23	23	27	28	27
BrPDI9–1																							69	21	33	27	30	22	24	22	35
BrPDI9–2																								32	23	24	27	21	22	18	31
BrPDI10–1																									**83**	**83**	34	29	40	44	27
BrPDI10–2																										**80**	35	27	27	41	28
BrPDI10–3																											36	29	29	23	25
BrPDI10–4																												26	30	30	27
BrPDI11–1																													**92**	75	**84**
BrPDI11–2																														75	**83**
BrPDI11–3																															75

Duplications are given bold

**Fig. 5:**
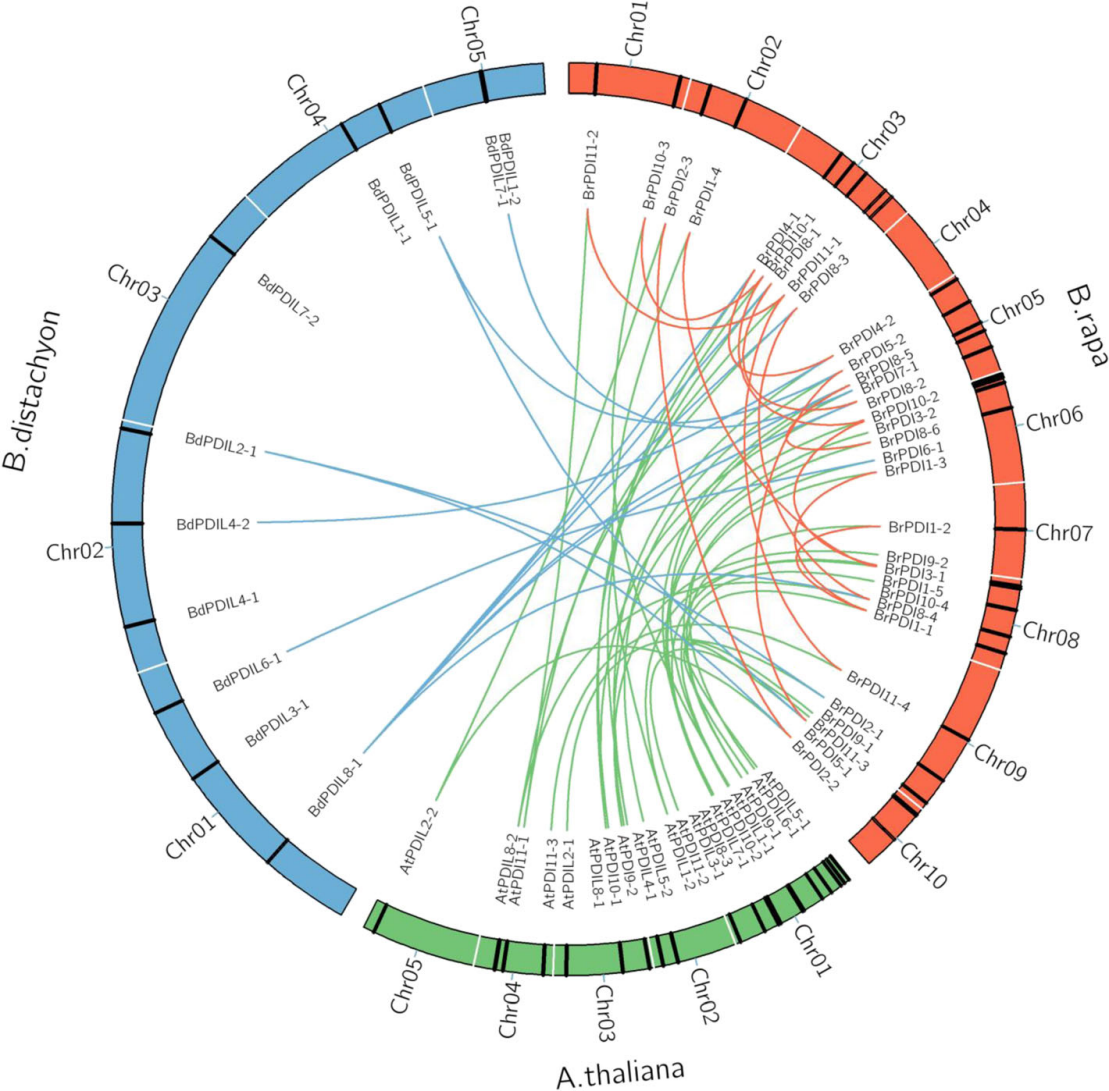
Microsynteny analysis of *PDI* genes among *B. rapa, Brachypodium distachyon* and *A. thaliana*. The chromosomes from the three species are indicated in different colors, orange, green and blue colors represent *B. rapa, A. thaliana* and *B. distachyon* chromosome respectively. Orange lines depiction of duplicated *BrPDI* genes on 10 *B. rapa* chromosomes

Three fractionated subgenomes, like least fractionated (LF), medium fractionated (MF1), and most fractionated (MF2) subgenomes are found in *B. rapa* genome. Notably, 32 *BrPDI* genes are distributed onto ten chromosomes with fractionation into three subgenomes, of which are 15 LF (46.87%), 8 MF1 (25%), and MF2 9 (28.13%; Fig. 4c and Table 1). In addition, 32 *BrPDI* genes are distributed in different block during evolution. Among them, 7 genes (22.58%) belong to AK8 block, followed by 19.45% of *BrPDI* genes into AK1, AK3 blocks; while only 6.45% of *BrPDI* genes were allocated to AK2, AK4, AK5 and AK6 (Fig. 4d). We have also found a total of 17 segmental duplicated *PDI* genes pairs in *B. rapa* genome (Fig. 5 and Table 3). Furthermore, the substitution rate of non-synonymous (Ka) and synonymous (Ks) ratios (Table 3) were calculated to assess the selection pressures among duplicated *BrPDI* gene pairs. In these analysis, Ka/Ks ratios were identified as lower than 1, 1 and above 1 indicating negative or purifying selection, neutral selection and positive selection, respectively. Nine out of seventeen *BrPDI* duplicated gene pairs, had Ka/Ks value below 1 (purifying selection) and rest of them had Ka/Ks value above 1(positive selection). Moreover, the estimated divergence time of *BrPDI* genes showed that duplication event started at 29.74 million years ago (MYA) and continued up to 1.62 MYA (Table 3).

**Table 3:** Estimated Ka/Ks ratios of the duplicated *BrPDI* genes with their divergence time in Chinese cabbage

Duplicated gene pairs			Ks	Ka	Ka/Ks	Duplication type	Types of selection	Time (mya)
BrPDI1–1	vs.	BrPDI1–2	0.0569	0.1138	2.0000	Segmental	Positive	1.89
BrPDI1–1	vs.	BrPDI1–3	0.1064	0.0726	0.6823	Segmental	Purifying	3.54
BrPDI1–2	vs.	BrPDI1–3	0.1113	0.0715	0.6424	Segmental	Purifying	3.71
BrPDI1–4	vs.	BrPDI1–5	0.2421	0.0349	1.4415	Segmental	Positive	8.07
BrPDI2–2	vs.	BrPDI2–3	0.0486	0.1554	3.1975	Segmental	Positive	1.62
BrPDI3–1	vs.	BrPDI3–2	0.1203	0.0698	0.5802	Segmental	Purifying	4.01
BrPDI4–1	vs.	BrPDI4–2	0.1093	0.1515	1.3860	Segmental	Positive	3.64
BrPDI5–1	vs.	BrPDI5–2	0.1010	0.4750	4.7029	Segmental	Positive	3.37
BrPDI8–1	vs.	BrPDI8–2	0.1773	0.0527	0.2972	Segmental	Purifying	5.91
BrPDI8–3	vs.	BrPDI8–4	0.1179	0.0343	0.2909	Segmental	Purifying	3.93
BrPDI8–5	vs.	BrPDI8–6	0.8922	0.3621	0.4058	Segmental	Purifying	29.74
BrPDI10–1	vs.	BrPDI10–2	0.2892	0.1337	0.4623	Segmental	Purifying	9.64
BrPDI10–1	vs.	BrPDI10–3	0.4183	0.2768	0.6613	Segmental	Purifying	13.94
BrPDI10–2	vs.	BrPDI10–3	0.7166	0.2026	0.2827	Segmental	Purifying	23.89
BrPDI11–1	vs.	BrPDI11–2	0.0895	0.1179	1.3173	Segmental	Positive	2.98
BrPDI11–1	vs.	BrPDI11–4	0.0969	0.2040	2.1052	Segmental	Positive	3.23
BrPDI11–2	vs.	BrPDI11–4	0.0985	0.3570	3.6243	Segmental	Positive	3.28

*Ks* the number of synonymous substitutions per synonymous site, *Ka* the number of nonsynonymous substitutions per nonsynonymous site, *MYA* million years ago

### Motif composition and intron–exon analysis

The intron–exon structures were almost consistent among the groups of *BrPDI* genes. Group I contained nine to ten exons; whereas, members of group V contained nine exons (Additional file 1: Figure S1). All members of group II possessed 10 to 11 exons, while members of group IV and IX contained 11 exons, and the member of group VIII composed with the highest number (15) of exons. Member of groups X and XI possessed 3–4 exons. Finally, member of groups VI and VII contained four and five exons, respectively. Conserved motifs among BrPDI proteins were investigated using the MEME motif search tool. Motifs 1 and 2 contained-CXXC- catalytic sites, which are necessary for isomerase and redox activity. Motifs 3 was present in all groups, but motifs 2 was absent in groups VI, VII, VIII, IX and X. Motif 1 and 5 were absent in group IV and XI, respectively. Relatively less conserved motif 6 was found only in groups I, II, III and VII. Motifs 4, 7, 8 and 9 were unique to group VIII and motif 10 was present in group VIII and XI only (Additional file 1: Figure S2).

### Microsyntenic analysis

A microsynteny map was constructed to find out orthologous gene pairs of *PDI* genes among *B. rapa*, *B.distachyon* and *A. thaliana* for exploration of evolutionary history and relationships among the genomes (Fig. 5). We found 22 orthologous gene pairs between *B. rapa* and *A. thaliana*; whereas, 13 orthologous gene pairs were identified between *B. rapa* and *B. distachyon* (Fig. 5). We found 17 duplicated PDI gene pairs in *B. rapa* genome. All of the segmental duplications are denoted with an orange line in fig. 5, no tandem duplications were found in *PDI* genes in *B. rapa*.

### Microarray data analysis

**Fig. 6:**
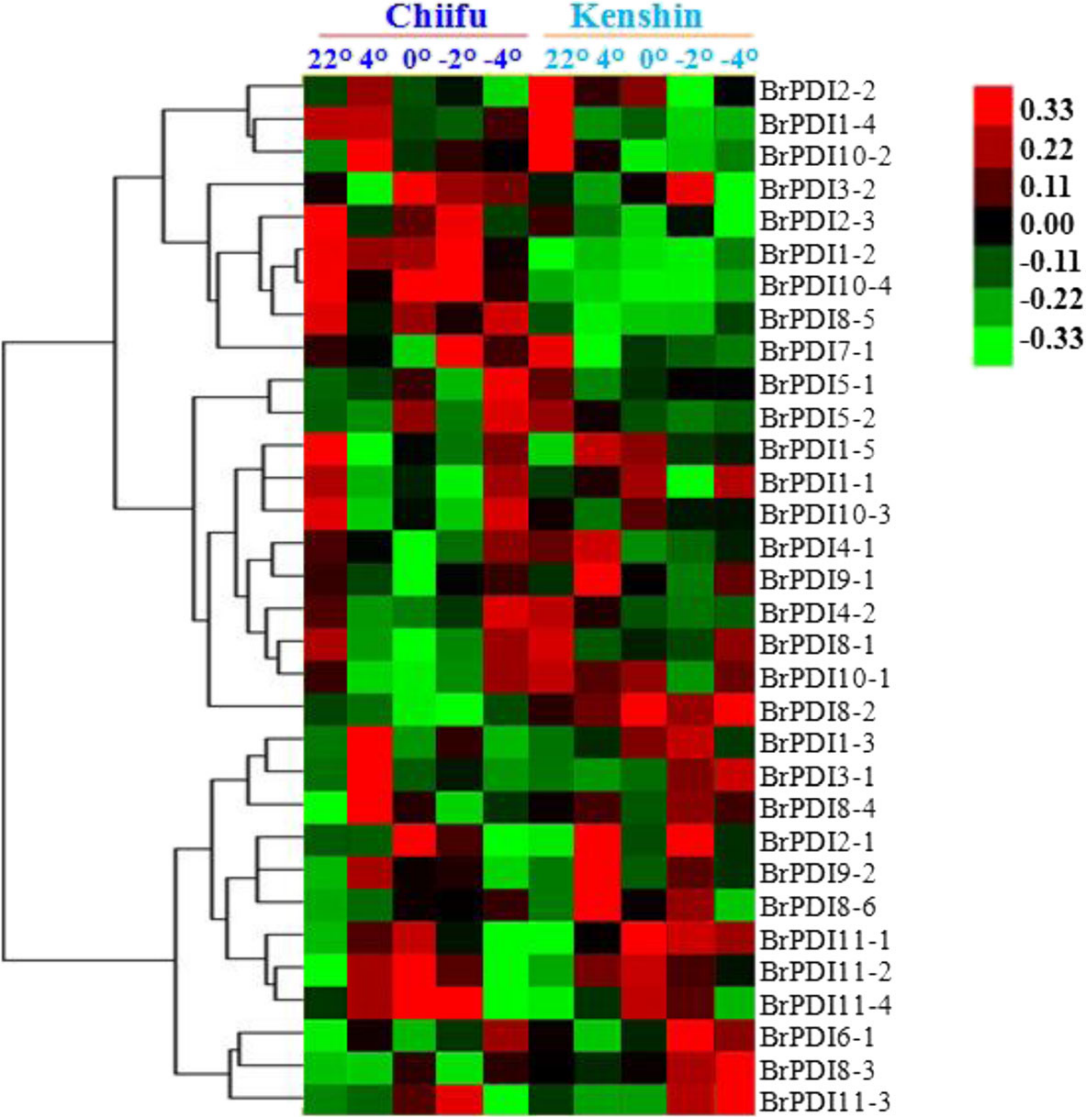
Heat map showing microarray expression of 32 *B. rapa PDI* genes against cold and freezing treatments in two inbred lines Chiifu and Kenshin

We used our previously published microarray data set to analyze the expression patterns of 32 *BrPDI* genes using two contrasting inbred lines ‘Chiifu’ and ‘Kenshin’, treated with cold and freezing stress (4 °C, 0 °C, −2 °C and −4 °C) for 2 h [20]. All of the *BrPDI* genes were differentially expressed in response to cold or freezing stress in two lines (Fig. 6). In Chiifu, most of the *BrPDI* genes were highly expressed upon exposure to cold and freezing temperatures. While, *BrPDI2–1, 4–1*, *8–2, 8–6, 9–1* and *10–1* genes showed higher expression during cold stresses in ‘Kenshin’ compared to ‘Chiifu’.

### Promoter analysis of *BrPDI* genes

*PDI* genes of Chinese cabbage might contain biotic and abiotic stress-responsive *cis*-acting elements in their promoter regions. Most of the *BrPDI* genes bear cold-responsive LTR *cis*-acting element, drought inducible MBS *cis*-element, and salt- or environmental stresses *cis*-acting element (WBOX and TC-rich repeats; Additional file 2: Table S1). The genes *BrPDI2–1, BrPDI2–2, BrPDI2–3, BrPDI3–2, BrPDI6–1, BrPDI7–1, BrPDI8–1, BrPDI8–2, BrPDI8–4, BrPDI10–1, BrPDI10–2, BrPDI10–3, BrPDI10–4, BrPDI11–1, BrPDI11–2, BrPDI11–3,* and *BrPDI11–4* have no ABA-responsive CE3 *cis*-acting element in their promoter regions. Majority of *BrPDI* genes contained more than one disease resistance or defense responsive *cis*-acting element (Box-W1, WBOX, and TC-rich repeats) except *BrPDI8–5, BrPDI10–3, BrPDI11–3* and *BrPDI11–4*.

### Organ-specific gene expression

**Fig. 7:**
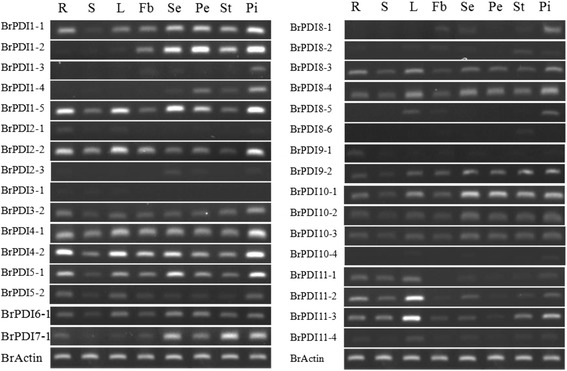
Expression profiles of *BrPDI* genes in various tissues, as determined by RT-PCR analysis. Eight amplified bands (from left to right) per gene represent amplified products from R- roots; S- stems, L- leaves; Fb- flower buds; Se- sepals; Pe- petals; St- stamens and Pi- pistils

We have used gene-specific primers of 32 *BrPDIs* for RT-PCR to check the expression patterns of *BrPDI*s in different organs (root, stem, leaf, flower buds, sepals, petals, stamens, and pistils) in Chinese cabbage line SUN-3061. Semi-quantitative RT-PCR analysis revealed that among the 32 genes, 15 were ubiquitously expressed in all organs (Fig. 7). Three genes (*BrPDI 1–2, 9–1* and *11–4*) were slightly expressed in all of the tested organs. *BrPDI 7–1* was highly expressed in floral parts but slightly expressed in roots, stems, leaves and flower buds. On the other hands, *BrPDI 1–2, 1–3, 1–4, 2–3* and *8–1* were totally absent in roots, stems and leaves but only expressed in flower buds and floral parts. In addition, *BrPDI 2–1* was weakly expressed in roots, leaves and pistil; while *BrPDI3–1* was expressed slightly in roots, stems, and leaves. *BrPDI8–5* was expressed in leaves, flower buds, and pistils; the *BrPDI 8–6* was expressed in stamens only*. BrPDI10–4* was appeared with very low signal in leaves, sepals and pistils. *BrPDI 11–1* and *BrPDI1 11–2* were highly expressed in vegetative organs but weekly expressed in reproductive organs (Fig. 7).

### Analysis of abiotic stress-responsive gene expression

**Fig. 8:**
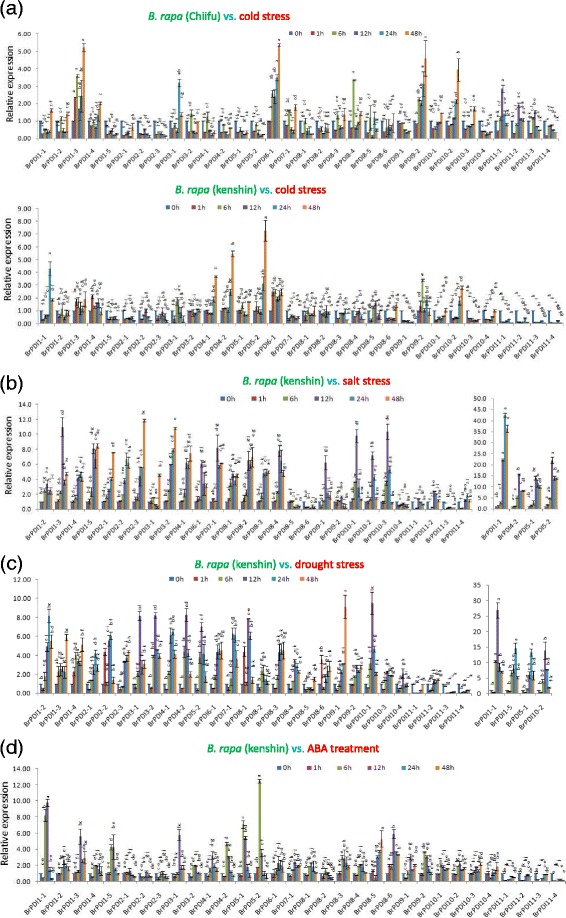
Real-time PCR expression analysis of *BrPDI* genes after treatment with (**a**) cold, (**b**) salinity, (**c**) drought, (**d**) ABA. The error bars represent the standard error of the means of three replications

Gene-specific primers of *BrPDI* genes were used to obtain expression profiles in response to various abiotic stresses. Expression patterns of *BrPDI* genes in response to cold was obtained by analyzing qPCR data from cold treated two contrasting Chinese cabbage lines, ‘Chiifu’ and ‘Kenshin’. In addition, we have calculated the expression patterns of different *BrPDI* genes during salt, drought and ABA stresses using qPCR data of Chinese cabbage line ‘kenshin’. Most of the *BrPDI* genes were significantly down regulated over the time course of cold stress, while six genes of ‘Chiifu’ (*BrPDI 1–3, BrPDI 3–1, BrPDI 6–1, BrPDI 9–2, BrPDI 10–2* and *BrPDI 11–1*) were significantly up-regulated on the advancement of cold stress durations (Fig. 8a). By contarst, most of the *BrPDI* genes in ‘Kenshin’ were significantly down-regulated throughout the stress period, however, only four genes (*BrPDI 1–1, BrPDI 4–1, BrPDI 4–2,* and *BrPDI 5–2*) showed high expression in ‘Kenshin’ compared to ‘Chiifu’ (Fig. 8a).

High transcript levels were detected in 24 *BrPDI* genes over time courses and these genes were up-regulated in response to salt stress. *BrPDI 1–1* gene was exhibited approximately 42- fold higher expression at 24 h*,* whereas *BrPDI 4–2, BrPDI 5–1* and *BrPDI 5–2* were exhibited 15-, 13-, and 19-fold higher expression, respectively than control at 12 h time point (Fig. 8b). In addition, *BrPDI 1–3*, *BrPDI 6–1, BrPDI 7–1, BrPDI 8–4, BrPDI 9–1, BrPDI 10–1, BrPDI 10–2*, and *BrPDI 10–3* were significantly up-regulated up to 12 h time point (Fig. 8b). Besides, *BrPDI 1–4, BrPDI 1–5, BrPDI 2–1, BrPDI 2–3, BrPDI 3–2, BrPDI 4–1* and *BrPDI 8–3* were up-regulated approximately 5-, 8-, 7-, 11-, 11-, 7-, and 5-fold, respectively at 48 h.

In drought stress, most of the *BrPDI* genes were up-regulated at 12 h and 24 h; then gradually down-regulated with the advancement of time courses (Fig. 8c). Eight genes (*BrPDI 1–1, BrPDI 3–1, BrPDI 3–2, BrPDI 4–2, BrPDI 5–2, BrPDI 8–1, BrPDI 10–1*, and *BrPDI 10–2)* were significantly up-regulated at 12 h. Five genes (*BrPDI 1–2, BrPDI 1–5, BrPDI 2–1, BrPDI 2–2,* and *BrPDI 5–1*) were significantly up-regulated at 24 h drought stress, thereafter being down-regulated (Fig. 8c). *BrPDI 1–1, BrPDI 1–5, BrPDI5–1* and *BrPDI 10–2* exhibited about 27-, 15-, and 14- fold higher expression, respectively compared to control. Most of the *BrPDI* genes had low level of expression in response to ABA treatment (Fig. 8d). *BrPDI 1–1, BrPDI 1–3, BrPDI 1–5, BrPDI 3–1, BrPDI 4–2, BrPDI 5–1,* and *BrPDI 5–2* were significantly up-regulated at 6 h and 12 h time point. However, those *BrPDI* genes were down-regulated at the beginning of ABA stress, but start up-regulation at 6 h and continued up to 12 h time point, thereafter gradually down-regulated over the remaining time courses. Four genes (*BrPDI 11–1, BrPDI 11–2, BrPDI 11–3 and BrPDI 11–4*) were down-regulated over the time courses. *BrPDI 1–1* gene was 10-fold up-regulated at 12 h; while *BrPDI 5–*2 gene showed 13-fold up-regulation at 6 h time points compared to control (Fig. 8d).

### Expression profiling of the *BrPDI* genes during chilling injury treatment

**Fig. 9:**
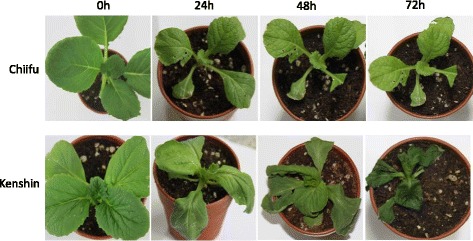
Comparative chilling injury symptoms in cold tolerant ‘Chiifu’ and ‘Kenshin’ plant at different time points (0 h, 24 h, 48 h, and 72 h) against chilling treatment (0 ° C). Chilling injury first appeared at 24 h in ‘Kenshin’ thereby gradually progress with the advancement of time points. The ‘kenshin’ plants are completely chill injured at 72 h time point but no chilling injury symptoms appeared in ‘Chiifu’ plants at any time points

**Fig. 10:**
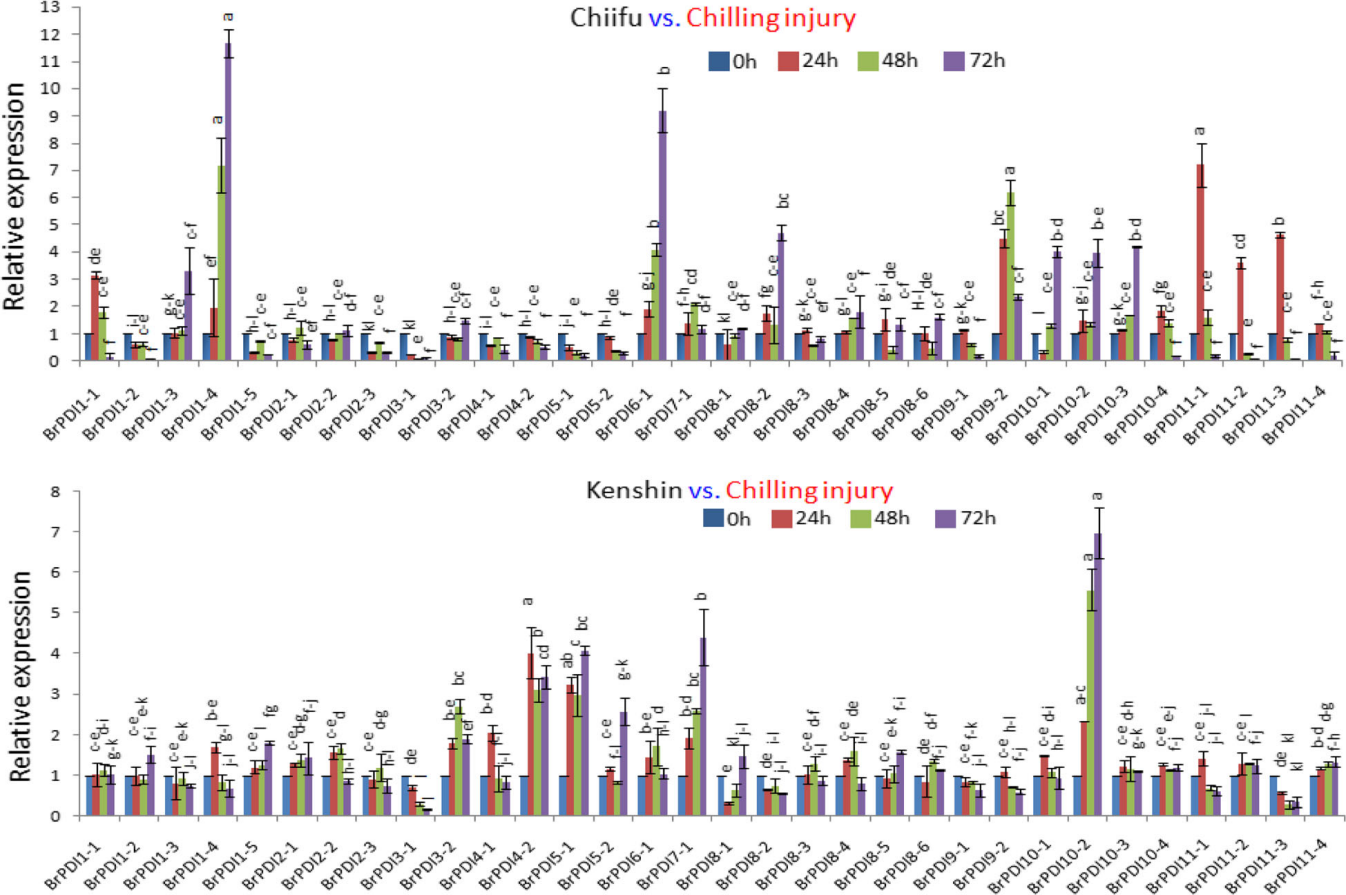
Real-time PCR expression analysis of *BrPDI* genes after treatment with Chilling injury. The error bars represent the standard error of the means of three replications

Chilling injury experiment with cold tolerance ‘Chiifu’ and cold sensitive ‘Kenshin’ was conducted to get the deep insight of the expression of the target cold tolerance gene as an alternative of functional analysis of the predicted genes. We exposed the ‘Chiifu’ and ‘Kenshin’ seedlings in cold injury treatment at 0 °C for 72 h until complete chilling injury of the susceptible (Kenshin). First remarkable chilling injury was recognized in ‘Kenshin’ seedlings at 24 h time point and gradually progress with the enhancement of time courses, ‘Kenshin’ seedlings were completely injured at 72 h time course. Whereas, no chilling injury was observed in ‘Chiifu’ seedlings up to 72 h time point, but plant growth become stunted (Fig. 9). In ‘Chiifu’, eight genes showed higher transcript level. *BrPDI1–4* and *BrPDI6–1* genes showed significant up-regulation about 12- and 9-fold higher expressions, respectively at 72 h time point in ‘Chiifu’ compared to ‘Kenshin’ (Fig. 10). Moreover, *BrPDI9–2* and *BrPDI11–1* showed about 6- and 8-fold higher expressions at 48 h and 24 h time point, respectively. In addition, *BrPDI10–2* gene exhibited higher expression in both line at 72 h time point. In ‘kenshin’, four genes (*BrPDI4–2, BrPDI5–1, BrPDI5–2*, and *BrPDI7–1*) showed higher expression against chilling injury treatment (0 °C).

### Analysis of biotic stress-related gene expression

**Fig. 11:**
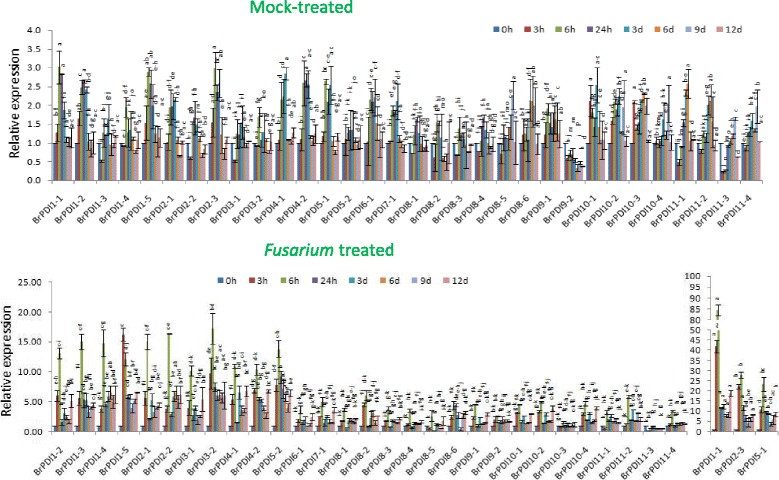
Real-time PCR expression analysis of *BrPDI* genes after treatment with *Fusarium oxysporum* f.sp*. conglutinans.* The error bars represent the standard error of the means of three replications

To elucidate the expression patterns of *BrPDI* genes in response to *Fusarium oxysporum* f. sp. *conglutinans* infection, we have collected leaf samples from infected and mock-treated Chinese cabbage line ‘Chiifu’ for performing qPCR. This fungus causes wilt and root rot diseases in *Brassica* crops. The *BrPDI* genes exhibited distinct expression patterns in response to *Fusarium* infection. Out of 32 genes, 14 showed high expression at 6 h time point (Fig. 11). The expression levels of *BrPDI 1–1, BrPDI 2–3,* and *BrPDI 5–1* were approximately 27-, 10-, and 11-fold higher, respectively compared to mock-treated plants. Fifteen genes (*BrPDI 6–1, BrPDI 7–1, BrPDI 8–1, BrPDI 8–2, BrPDI 8–3, BrPDI 8–4, BrPDI 8–5, BrPDI 8–6, BrPDI 9–1, BrPDI 10–1, BrPDI 10–2, BrPDI 10–4, BrPDI 11–1, BrPDI 11–2*, and *BrPDI 11–4*) had very little or insignificant expression compared to mock-treated (uninfected) plants. However, *BrPDI10–3* and *BrPDI11–3* showed no response against *F. oxysporum* infection.

## Discussion

In genome-wide exploration study of Chinese cabbage *PDI* genes; we have identified 32 *PDI* genes identical to 21 *PDI* genes of *Arabidopsis*. The Chinese cabbage genome has gone under two duplication events since its divergence from *Arabidopsis* [21]. The evolutionary relationship between *Arabidopsis* and Chinese cabbage is also supported by our results. We analyzed 32 *BrPDI* genes expression pattern based on the microarray data set in two contrasting Chinese cabbage inbred lines, ‘Chiifu’ and ‘Kenshin’ exposed to cold and freezing stress (4 °C, 0 °C, −2 °C, and −4 °C) [20]. From an evolutionary point of view, gene duplications, tandem and segmental duplications can increase the gene numbers of a particular gene family [22]. Distribution of *BrPDI* genes in the *B. rapa* genome are also affected by segmental duplication, tandem duplication, polyploidization etc. during evolution [23, 24]. In addition, genome triplication events of *B. rapa* may also be played important role in the expansion of *PDI* gene family*.* We found 17 pairs of segmental duplicated *PDI* genes in Chinese cabbage genome (Fig. 5 and Table 3), this result indicates that the expansion of *PDI* gene family in *B. rapa* genome depicted on segmental gene duplication.

We also investigated evolutionary history of *BrPDI* gene family and calculated the Ka (the number of synonymous substitutions per synonymous site), Ks (the number of nonsynonymous substitutions per nonsynonymous site) and Ka/Ks ratios of segmental duplicated gene pairs. Nine gene pairs had Ka/Ks < 1 (purifying selection) and eight gene pairs had Ka/Ks >1(positive selection; Table 3) indicating purifying selection and positive selection together accelerated evolution and functional divergence of *BrPDI* genes in *B. rapa* genome. Based on the synonymous substitution rate, we calculated divergence time of the *BrPDI* genes and found that duplication started on 29.74 MYA and stop at 1.62 MYA (Table 3) which indicate *PDI* genes divergence take place after the genome triplication events in *B. rapa.* Genome triplication event of *B. rapa* has occurred from the ancestor of *A. thaliana* between five to nine million years ago (MYA; [25]). Besides this, the exons and introns distribution of *BrPDI* genes are also consistent with the number of exons and introns of *PDI* genes in *Arabidopsis*, which indicate their close evolutionary relationship. In addition with, by drawing microsyntenic map, we conclude that *BrPDI* genes are strongly related to those of *B. distachyon* and *A. thaliana.*

The Chinese cabbage *PDI* genes appeared as functionally differentiated for short time, and keeps as functional genes in the genome and maintaining complex functions in organ development. The features and functions of *PDI* family genes have been extensively studied in *Triticum aestivum* [14] and *Brachypodium distachyon* [15]. Our identified and characterized *PDI* genes in Chinese cabbage help to increase the understanding of role of this family gene. Most of the *BrPDI* genes were predominantly expressed in all organs suggested their functions in regulation of plant growth and development. Based on our organ-specific expression analysis, all *BrPDI* genes are expressed at least in one organ at variable level. *BrPDI 2–1* and *BrPDI 9–1* were highly expressed in root, which is consistent with the previous findings [14, 15]. In addition to their involvement in plant growth and development, *PDI* genes are also responsive to adverse environmental conditions [15]. We have found *BrPDI 1–2, BrPDI 1–3, BrPDI1–4, BrPDI 2–3 BrPDI 8–1* and *BrPDI 8–6* were expressed in flower buds and floral parts, suggesting their possible role in flower development.

The expression patterns of 32 *BrPDI* genes were analyzed using a whole-genome microarray dataset of two inbred lines of *B. rapa*, ‘Chiifu’ and ‘Kenshin’ imposed to various temperature treatment [26]. Thereof, we have selected *BrPDI* family genes for analyzing their differential expression patterns compared to control by constructing heat-map (Fig. 6). Our results suggest that *BrPDI* genes play a vital role against abiotic stress responses in Chinese cabbage*.* In addition, our main objectives were to identify putative candidate *PDI* genes related to stress response during plant growth and development. It is established fact that environmental stresses like, cold, salt, and drought are severely affected crop production and make threaten in food security worldwide. Therefore, application of artificial stress treatment helps to know, how plants adapt to stresses via molecular, morphological, and physiological mechanisms. There is intense interest in identifying stress-responsive genes and elucidate them to develop stress-tolerant crop cultivars. Relative expression of PDI family genes were estimated from the sample of the plants subjected to abiotic stresses (cold, salt and drought), hormone (ABA), chilling injury, and biotic (*F. oxysporum* f.sp.*conglutinans*) stress treatment over different time periods (Fig. 8a–d, 9~11) using qPCR data. In plants, PDIs are localized to the ER and other cellular compartments, such as nucleus and chloroplasts [27, 28]. Proteins in the ER of plants can lead to unfolding or misfolding due to complex physiological processes during different environmental stresses [29]. Plants possess multiple mechanisms for ensuring correct folding of proteins. PDI was the first reported catalyst of protein folding (2). In the current study, *BrPDI1–3, BrPDI3–1, BrPDI6–1, BrPDI9–2, BrPDI10–2*, and *BrPDI11–1* were highly expressed in cold-tolerant ‘Chiifu’ than in cold-susceptible ‘Kenshin’ during cold stress treatment. *PDI* genes that are highly expressed under cold stress may encode proteins catalyze for breakage of disulfide bonds in misfolded proteins, restoring their proper functioning through correction of folding patterns. Lu & David [30] reported six *Arabidopsis* genes; those were up-regulated due to chemicals such as dithiothreitol (DTT), β-mercaptoethanol and tunicamycin induction. In current study, we have found *BrPDI1–1, BrPDI4–1, BrPDI4–2,* and *BrPDI5–2* were highly expressed under cold stress in cold-susceptible ‘Kenshin’ compared to cold-tolerant ‘Chiifu’, suggesting that these four genes might be related to the cold-susceptibility in ‘Kenshin’. However, *BrPDI2–1, BrPDI2–2, BrPDI5–2, BrPDI8–1, BrPDI8–6, BrPDI9–1, BrPDI10–4*, and *BrPDI11–4* were not expressed or even down-regulated in ‘Chiifu’ under cold treatment confirmed that these genes contain putative cold-inducible LTR *cis*-acting elements. Notably, these genes also contain a heat-inducible HSE *cis*-acting element. Hence, both cold- and heat-inducible *cis*-acting elements are presented in the promoter regions of the same genes suggesting that the latter *cis*-acting element may down-regulate these genes under cold stress. Two contrasting cold tolerant Chinese cabbage lines were used in this study to predict the putative cold susceptible and cold tolerant *BrPDI* gene(s) of this crop. Four *BrPDI* genes (*BrPDI1–4*, *BrPDI6–1*, *BrPDI9–2*, and *BrPDI11–1*) exhibited higher expression in ‘Chiifu’ compared to ‘Kenshin’ at the level of chilling injury. Among these four genes *BrPDI6–1*, *BrPDI9–2*, and *BrPDI11–1* genes were common in the higher expression in cold stress and chilling injury level treatments revealed their active involvement to overcome the cold and chilling injury at different time points of the cold tolerant line ‘Chiifu’. Therefore, from the in depth expression data, it is clearly evident that *BrPDI6–1*, and *BrPDI9–2* genes are the putative candidate in Chinese cabbage for overcoming cold and even chilling injury temperature (0 ° C).

With few exceptions, *BrPDI* genes are highly expressed in Chinese cabbage under salt and drought stress, among them *BrPDI1–1* to *BrPDI5–2* genes encode the proteins which containing two active sites and a C-terminal ER retention signal composed of four amino acids (KDEL/ VASS/ MADD), might be involved in enhancing protein-folding in the ER. *BrPDI1–1, BrPDI1–3, BrPDI1–5, BrPDI3–1, BrPDI4–2, BrPDI5–1, BrPDI5–2, BrPDI8–5*, and *BrPDI8–6* were highly expressed under ABA stress compared to control. Mittler et al. [31] and Sakamoto et al. [32] observed rapid accumulation of reactive oxygen species (ROS) in plant cells subjected to environmental stresses such as salt, drought, and ABA. Indeed, during environmental stress (e.g., drought, salt, ABA, UV, and heat exposure) ROS levels can increase dramatically, which may significantly damage cell structure. PDI proteins play a significant role in thioredoxin-based redox pathway, which comprises part of the antioxidative defense system [33]. Abiotic stress usually leads to protein unfolding, misfolding, and aggregation; which represent a common threat to living cell [34]. Efficient protein repair systems and/or protein folding stability helps plant to survive in adverse environmental conditions [35]. Most *BrPDI* genes were differentially expressed in response to salt, drought, and ABA treatment, which is consistent with the findings of Zhu et al. [15]. Those genes are highly expressed in response to salt, drought, and ABA might function in the repair of misfolded proteins, thereby playing a role in plant resistance to the stresses. The up-regulated genes contain the respective stress-responsive *cis*-elements in their promoter regions (Additional file 2: Table S1).

*PDI* genes have been previously shown to be contributed against powdery mildew resistance in common wheat [36]. In this study, we have analyzed expression profiles of these genes in response to *Fusarium oxysporum* f.sp. *conglutinans* infection. Moreover, 14 genes were up-regulated compare to mock treated one due to infection of fungal pathogen at 6 h time point, whose genes containing a disease resistance Box-W1 *cis*-acting element (Fig. 11, Additional file 2: Table S1). By contrast, *BrPDI 6–1~ BrPDI 11–4* do not contain the Box-W1 element, those exhibited almost no expression due to fungal infection. Thus, the highly expressed *BrPDI* genes might play a vital role in response against fungal pathogen. Therefore, this might be the putative candidate genes for developing *Fusarium oxysporum* f.sp. *conglutinans* resistant Chinese cabbage cultivars through marker assisted back crossing (MAB) or gene technology. Besides, the up-regulated *BrPDI* genes might play differential roles in signal transduction pathways and/or cooperate with other genes to form networks for defending plants against adverse environmental conditions.

## Conclusion

In conclusion, this is the first report of genome-wide characterization of PDIs in Chinese cabbage. We identified 32 *BrPDI* genes in the Chinese cabbage genome and characterized them based on motif distribution, protein structure, classification, number of introns -exons, sequence similarities, and expression patterns in response to abiotic (cold, salt, and drought) stresses, ABA treatment and biotic stress (*F. oxysporum* f.sp. *conglutinans* inoculation). We have also recognized *BrPDI* genes may play roles in biotic and abiotic stress responses. Several *BrPDI* genes (*BrPDI1–1, BrPDI1–4, BrPDI1–5, BrPDI3–1, BrPDI3–2, BrPDI4–1, BrPDI4–2, BrPDI5–1, BrPDI5–2, BrPDI6–1, BrPDI7–1, BrPDI8–2, BrPDI9–1, BrPDI9–2*, and *BrPDI11–1*) might function in responses to multiple stresses. The identified stress-induced *BrPDI* genes help to elucidate the complex regulatory network underlying stress resistance mechanisms. In addition, these genes represent a useful resource to molecular breeders for marker assisted back crossing (MAB) and/or engineering transgenic plants in future research with increased resistance to biotic and abiotic stresses.

## Methods

### Identification and sequence analysis of *BrPDI* genes

Chinese cabbage PDI family members were identified using the SWISSPROT tool of the *B. rapa* database (http://brassicadb.org/brad/searchAll.php; 20) and NCBI using the keyword “PDI”. We also searched these genes from a microarray Br135K dataset of two contrasting Chinese cabbage inbred lines, Chiifu and Kenshin exposed to low-temperature stress. The *Arabidopsis* PDI sequences were used as the query to perform a BLAST search setting a cutoff e-value of <10^−10^. The CDS (coding DNA sequences) and protein sequences of the identified PDIs were obtained from the *B. rapa* genomic database. The PDI protein sequences were further analyzed for the presence of a thioredoxin domain using the web tool “SMART” (http://smart.embl-heidelberg.de/ [37]) and NCBI (https://www.ncbi.nlm.nih.gov/cdd). Additionally, the primary structures of the genes (protein length, molecular weight and isoelectric point) were analyzed using ExPasy (http://web.expasy.org/compute_pi/). ORFs were identified using ORF finder at NCBI (https://www.ncbi.nlm.nih.gov/orffinder/). Sequence alignment of PDI proteins were carried out using CLUSTAL Omega (http://www.ebi.ac.uk/Tools/msa/clustalo/). Sub-genome fractionation and positional information for all idetified *BrPDI* genes on 10 chromosomes of *B. rapa* were retrieved from the *Brassica* database, and draft maps of the locations of the *BrPDI* genes were constructed using Map Chart version 2.30 (http://www.wageningenur.nl/en/show/Mapchart.htm ). The Gene Structure Display Server (GSDS) web tool (http://gsds.cbi.pku.edu.cn/) was used to determine the number of introns and exons by aligning CDS and genomic sequences of the *PDI* genes [38]. Putative *cis*-acting regulatory elements in the *BrPDI* genes were predicted in regions of approximately1500-bp upstream of the translation initiation site [ATG] using the PlantCARE (http://bioinformatics.psb.ugent.be/webtools/plantcare/html/).

### Phylogenetic and conserved motif analysis of Chinese cabbage *PDI* genes

The predicted BrPDI protein sequences were aligned with those of *Arabidopsis*, *Brachypodium distachyon and Zea mays* and Chinese cabbage using Clustal Omega, and a phylogenetic trees was constructed based on the condensed alignment in MEGA6.06 using the Neighbor-Joining (NJ) algorithm [39], with the parameters set at 1000 replications for bootstrap values and complete deletion mode to analyze tree topology and reliability. The genes which were used in phylogenetic analysis, name and accession number provided in Additional file 2: Table S2. Motif analysis of proteins was performed using MEME (Expectation Maximization for Motif Elicitation v4.10.1) [40] with the following parameters: (1) number of motifs = 10, (2) Motif width ≥ 6 and ≤50.

### Chromosome localization and gene duplications

The physical locations of *BrPDI* genes were collected from *B. rapa* genomic database and the positions of the *BrPDI* genes were drafted to ten *B. rapa* chromosomes by map chart program. Duplicated *BrPDI* genes were identified using BLAST searched against themselves, notably identity and query coverage were >80% of those particular genes [41]. Tandem duplicated genes were marked as an array of two or more homologous genes within a distance of 100 kb. The synonymous rate (*Ks*), nonsynonymous rate (*Ka*), and evolutionary constraint (*Ka*/*Ks*) were calculated between the duplicated PDI gene pairs (Table 3), using the method of Nei & Gojobori [42] by Mega 6.0 software. The value of Ka/Ks ratio like >1, <1 and =1 are depicted for positive selection, purifying selection and neutral selection, respectively [43]. The divergence time was calculated using the formula T = *Ks*/2r Mya (Millions of years) where, *Ks* being the synonymous substitutions per site and r is considered 1.5 × 10–8 substitutions rate per site per year for dicot plants [44]. We reconstructed 24 conserved chromosomal blocks (labelled A–X) of *B. rapa* genome. Colour coding of the blocks was assigned based on their positions in a proposed ancestral karyotype (AK1–8) following the procedure stated by Cheng et al. [45] and Schranz et al. [46].

### Microarray and microsynteny of PDI gene family

Two contrasting inbred Chinese cabbage (*B. rapa* ssp. *pekinensis*) lines, ‘Chiifu’ and ‘Kenshin’ in respect to cold tolerance and cold susceptible, were used for microarray expression analysis Kayum et al. [47]. A heat map was generated based on transcript abundance value of *PDI* genes using Cluster 3.0 (http://bonsai.hgc.jp/~mdehoon/software/cluster/software.htm). The microsyntenic relationship of PDI genes among *B. rapa*, *Brachypodium distachyon* and *A. thaliana* were detected using Blast against whole genome of such crop species. Chromosomal positions of *PDI* genes were collected from respective databases and the relationship among the three crop species were plotted using circos software (http://circos.ca/) [48].

### Plant growth conditions, treatments and sampling

Surface sterilize seeds of two Chinese cabbage inbred lines ‘Chiifu’ and ‘Kenshin’ were grown in incubation room maintaining 24 °C temperature with 14/10 h (light/dark) condition at the Department of Horticulture, Sunchon National University, Korea. Abiotic stress treatments were imposed to four-week-old seedlings according to the methods described by Ahmed et al. [49], three biological replicates were maintained for abiotic stress treatments. Two contrasting Chinese cabbage inbred lines, cold tolerance ‘Chiifu’ and cold susceptible ‘Kenshin’ were used for abiotic stress cold and chilling injury stress experiment. Whereas, only ‘Kenshin’ was used to predict the *BrPDI* genes against other stress treatments (salt, drought, and ABA) due to unavailability of any contrasting genotypes for other abiotic stress treatments (salt, drought, and ABA) in our hand. By contrast, we assumed ‘Kenshin’ would be suitable for molecular characterizing of *BrPDI* genes for abiotic stress responsiveness rather than cold, because these type of genotypes are widely grown in tropics and sub-tropics. Fresh roots and leaves (third and fourth leaves) were excised from stress-treated plants over time points (0 h, 1 h, 4 h, 12 h, 24 h, and 48 h). Samples were immediately frozen in liquid nitrogen and stored at −80 °C for RNA extraction. For chilling injury experiment, the Chinese cabbage inbred line ‘Chiifu’ and ‘Kenshin’ plants were grown in culture room under a 16 h light photoperiod maintaining 24 °C. The three week old seedlings were transferred to incubator (TOGA clean system; model: TOGA UGSR01) maintained 0 °C temperature and keep the seedling until clear remarkable cold injury symptoms appeared. Cold treated plants leaves were excised at 0 h, 24 h, 48 h and 72 h time points with the progression of cold injury in cold susceptible line ‘Kenshin’ (Fig. 9), thereby leaves were immediately frozen in liquid nitrogen and stored at −80 °C for RNA extraction. Total five biological replications were maintained for chilling injury treatment. However, inbred line ‘Chiifu’ was used for organ-specific expression profiling of *BrPDI* genes and also infection with *F. oxysporum* f. sp. *conglutinans* for biotic stress as described by Ahmed et al. [50]. *Fusarium*- and mock-infected leaves (4th and 5th leaves) were collected at 0 h, 3 h, 6 h, 24 h, 3d, 6d, 9d, 12d after inoculation and immediately frozen in liquid nitrogen and stored at −80 °C for RNA extraction.

### RNA extraction and cDNA synthesis

Total RNA was extracted from the samples (roots and leaves) using an RNeasy mini kit (Qiagen, USA) following the manufacturer’s instructions. DNA contamination was removed using RNase-free DNase (Promega, USA) following manufacturer’s protocol. The extracted RNA was quantified by UV spectrophotometry at A260 using a NanoDropND-1000 and NanoDrop v3.7 software (Nano Drop Technologies, USA). Complementary DNA (cDNA) was synthesised from total RNA using a First-Strand cDNA synthesis kit (Invitrogen, Japan) following the manufacturer’s instructions.

### Qualitative and quantitative PCR expression analysis

Qualitative expression analysis was conducted using one-step Emerald Amp GT PCR Master Mix (Takara, Japan) by RT-PCR. Gene-specific primers for *BrPDIs* were used for RT-PCR and the *BrActin* gene of Chinese cabbage was used as an internal control. RT-PCR was performed using 1 μL of 50 ng template cDNA using master mix contained 10 pmol of each primer (forward and reverse), 9 μL sterile water and 8 μL Emerald mix in a total volume of 20 μL. The PCR conditions were initial denaturation 94 °C for 5 min followed by 30 cycles of denaturation at 94 °C for 30s, annealing at 58 °C for 30s and extension at 72 °C for 45 s, with a final extension at 72 °C for 5 min. The PCR products were visualized on 1.2% agarose gel.

Quantitative Real-time PCR (qRT-PCR) was conducted using 10 μL reaction volume consisted of 5 μL 2× Quanti speed SYBR mix, 1 μL (10 pmol) each of forward (F) and reverse (R) gene-specific primers (Additional file 2: Table S3), 1 μL template cDNA (50 ng) and 2 μL ultra-pure water (ddH_2_O). The conditions for real-time PCR was initial denaturation at 95 °C for 5 min, followed by 40 cycles of denaturation at 95 °C for 10 s, annealing at 58 °C for 10 s and extension at 72 °C for 15 s. The qRT-PCR reactions were normalized using the Chinese cabbage *Actin* gene as a reference for all comparisons [51]. Fluorescence was measured at last step of each cycle, and three replicates were used for each sample. Amplification detection and data were processed using the Light cycler® 96 SW 1.1 software and the cq value was calculated using the 2^-ΔΔ^C_T_ method to determine the relative expression. The relative expression data was statistically analyzed (Tukey HSD test) and lettering was done using Minitab 17 software (*https://www.* *minitab* *. com/products/* *minitab* */*).

## Additional files

Additional file 1: Figure S1. The genomic structures of *BrPDI* genes. Solid green boxes and red lines indicate exons and introns, respectively. The bottom scale indicates length of exons and introns. **Figure S2.** Schematic representation of motif compositions in the BrPDI protein sequences. Different motifs logo, numbered 1–10, motif are displayed in different colored boxes. The names of all members are displayed on the left-hand side. (PPT 932 kb)

Additional file 2: Table S1. Putative *cis*-elements, more than 6 bp, were identified in 32 *BrPDI* genes in Chinese cabbage. **Table S2.** A total of 76 *PDI* and *PDIL* genes name and accession numbers from 4 species used for constructing phylogenetic tree, including 32 from *Brassica rapa,*(*Br*) 21 from *Arabidopsis thaliana* (*At*)*,*11 from *Brachypodium distachyon* (*Bd*) and 12 from *Zea mays* (*Zm*). **Table S3.** Primers for RT-PCR andreal-time PCR analysis of 32 *BrPDI* genes (DOC 472 kb)

## Abbreviations

ABA: Abscisic acid
AK: Karyotype
At: *Arabidopsis thaliana*
Bd: *Brachypodium distachyon*
Br: *Brassica rapa*
BRAD: Brassica database
C: Chiifu
CDS: Coding DNA sequence
Cl: Cluster
dai: Days after infection
Er: Endoplasmic reticulum
*F.oxysporum*: *Fusarium oxysporum*
f.sp.: Fungal species
HTH: Helix-turn-helix
K: Kenshin
Ka: Nonsynonymous substitutions per nonsynonymous site
kb: Kilo basepair
Ks: Synonymous substitutions per synonymous site
LF: Least fractionated
MF1: Medium fractionated
MF2: Most fractionated
MYA: Million year
PD: Protein disulfide isomerase
qPCR: Quantitative polymerase chain reaction
RT-PCR: Reverse transcription polymerase chain reaction
TRX: Thioredoxin
Zm: *Zea mays*
