# Supplementary material for: Genome-wide characterization and expression profiling of PDI family gene reveals function as abiotic and biotic stress tolerance in Chinese cabbage (Brassica rapa ssp. pekinensis)
Source: BMC Genomics. 2017 Nov 16;18:885. doi: 10.1186/s12864-017-4277-2 (PMC5691835; doi:10.1186/s12864-017-4277-2)
Supplement: Supplementary file 1 — The genomic structures of BrPDI genes. Solid green boxes and red lines indicate exons and introns, respectively. The bottom scale indicates length of exons and introns. Figure S2. Schematic representation of motif compositions in the BrPDI protein sequences. Different motifs logo, numbered 1–10, motif are displayed in different colored boxes. The names of all members are displayed on the left-hand side. (PPT 932 kb) [file 12864_2017_4277_MOESM1_ESM.ppt]

## Slide 1
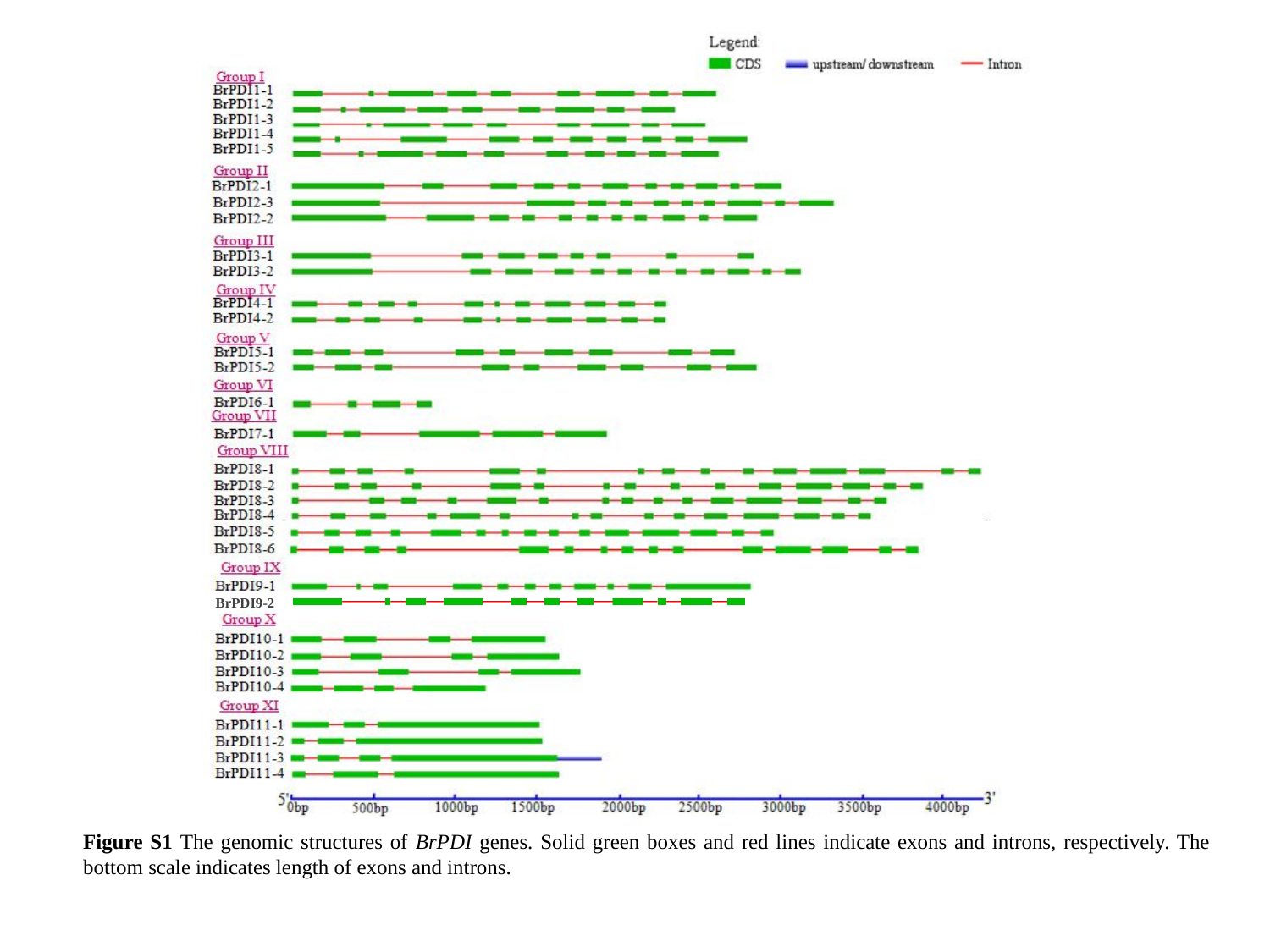

BrPDI9-2
Figure S1 The genomic structures of BrPDI genes. Solid green boxes and red lines indicate exons and introns, respectively. The bottom scale indicates length of exons and introns.

## Slide 2
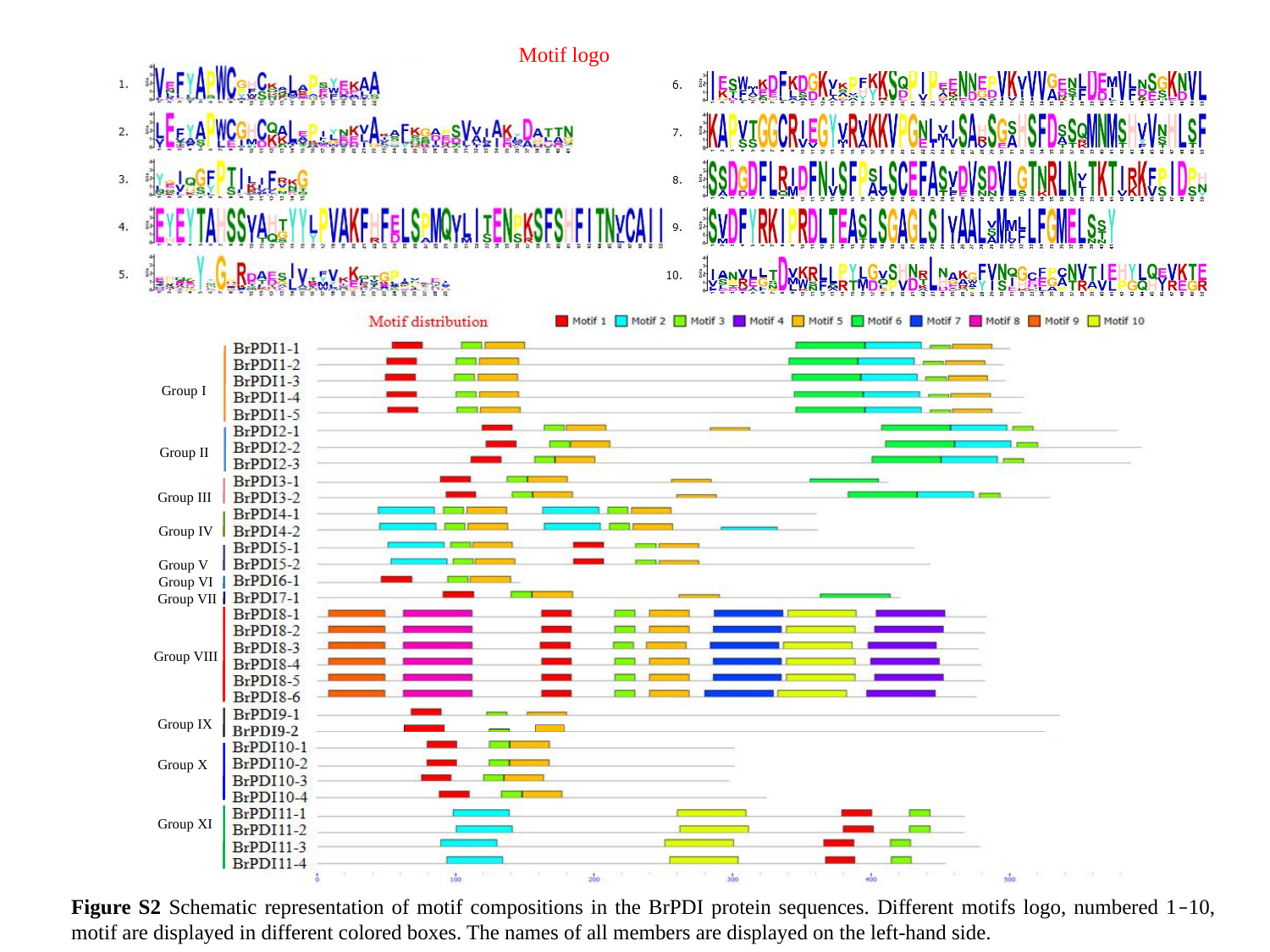

Motif logo
Group I
Group II
Group III
Group IV
Group V
Group VI
Group VII
Group VIII
Group IX
BrPDI9-2
Group X
Group XI
Figure S2 Schematic representation of motif compositions in the BrPDI protein sequences. Different motifs logo, numbered 1–10, motif are displayed in different colored boxes. The names of all members are displayed on the left-hand side.
