# Supplementary material for: Genome-wide characterization and expression profiling of PDI family gene reveals function as abiotic and biotic stress tolerance in Chinese cabbage (Brassica rapa ssp. pekinensis)
Source: BMC Genomics. 2017 Nov 16;18:885. doi: 10.1186/s12864-017-4277-2 (PMC5691835; doi:10.1186/s12864-017-4277-2)
Supplement: Supplementary file 2 — Putative cis-elements, more than 6 bp, were identified in 32 BrPDI genes in Chinese cabbage. Table S2. A total of 76 PDI and PDIL genes name and accession numbers from 4 species used for constructing phylogenetic tree, including 32 from Brassica rapa,(Br) 21 from Arabidopsis thaliana (At),11 from Brachypodium distachyon (Bd) and 12 from Zea mays (Zm). Table S3. Primers for RT-PCR andreal-time PCR analysis of 32 BrPDI genes (DOC 472 kb) [file 12864_2017_4277_MOESM2_ESM.doc]

**Table S1** Putative cis-elements, more than 6 bp, were identified in 32 *BrPDI* genes in Chinese cabbage

| Gene name | *Cis* element | Organism | Sequence | Function |
| --- | --- | --- | --- | --- |
| BrPDI1-1 | [ABRE](http://bioinformatics.psb.ugent.be/webtools/plantcare/cgi-bin/show_site_info.htpl?QWhere=ID_of_Site like 'AT~ABRE'&StartAt=0&NbRecs=10) | *Arabidopsis thaliana* | TACGTG | Cis-acting element involved in the abscisic acid responsiveness |
|  | [AAAC-motif](http://bioinformatics.psb.ugent.be/webtools/plantcare/cgi-bin/show_site_info.htpl?QWhere=ID_of_Site like 'SO~AAAC-motif'&StartAt=0&NbRecs=10) | *Spinacia oleracea* | CAACAAAAACCT | Light responsive element |
|  | [Box-W1](http://bioinformatics.psb.ugent.be/webtools/plantcare/cgi-bin/show_site_info.htpl?QWhere=ID_of_Site like 'PC~Box-W1'&StartAt=0&NbRecs=10) | *Petroselinum crispum* | TTGACC | Fungal elicitor responsive element |
|  | [CGTCA-motif](http://bioinformatics.psb.ugent.be/webtools/plantcare/cgi-bin/show_site_info.htpl?QWhere=ID_of_Site like 'HV~CGTCA-motif'&StartAt=0&NbRecs=10) | *H.vulgare* | CGTCA | Cis-acting regulatory element involved in the MeJA-responsiveness |
|  | [CE3](http://bioinformatics.psb.ugent.be/webtools/plantcare/cgi-bin/show_site_info.htpl?QWhere=ID_of_Site like 'OS~CE3'&StartAt=0&NbRecs=10) | *O. sativa* | GACGCGTGTC | Cis-acting element involved in ABA and VP1 responsiveness |
|  | [GARE-motif](http://bioinformatics.psb.ugent.be/webtools/plantcare/cgi-bin/show_site_info.htpl?QWhere=ID_of_Site like 'BO~GARE-motif'&StartAt=0&NbRecs=10) | *B. olerace* | TCTGTTG | Gibberellin-responsive element |
|  | HSE | *B. oleracea* | AGAAAATTCG | *Cis*-acting element involved in heat stress responsiveness |
|  | [LTR](http://bioinformatics.psb.ugent.be/webtools/plantcare/cgi-bin/show_site_info.htpl?QWhere=ID_of_Site like 'HV~LTR'&StartAt=0&NbRecs=10) | *Hordeum vulgare* | CCGAAA | Cis-acting element involved in low-temperature responsiveness |
|  | [MBS](http://bioinformatics.psb.ugent.be/webtools/plantcare/cgi-bin/show_site_info.htpl?QWhere=ID_of_Site like 'AT~MBS'&StartAt=0&NbRecs=10) | *A. thaliana* | CAACTG | MYB binding site involved in drought-inducibility |
|  | [TC-rich repeats](http://bioinformatics.psb.ugent.be/webtools/plantcare/cgi-bin/show_site_info.htpl?QWhere=ID_of_Site like 'NT~TC-rich repeats'&StartAt=0&NbRecs=10) | *Nicotiana tabacum* | ATTTTCTCCA | Cis-acting element involved in defense and stress responsiveness |
|  | [TGA-element](http://bioinformatics.psb.ugent.be/webtools/plantcare/cgi-bin/show_site_info.htpl?QWhere=ID_of_Site like 'BO~TGA-element'&StartAt=0&NbRecs=10) | *Brassica oleracea* | AACGAC | Auxin-responsive element |
| BrPDI1-2 | [Box-W1](http://bioinformatics.psb.ugent.be/webtools/plantcare/cgi-bin/show_site_info.htpl?QWhere=ID_of_Site like 'PC~Box-W1'&StartAt=0&NbRecs=10) | *P. crispum* | TTGACC | Fungal elicitor responsive element |
|  | [CGTCA-motif](http://bioinformatics.psb.ugent.be/webtools/plantcare/cgi-bin/show_site_info.htpl?QWhere=ID_of_Site like 'HV~CGTCA-motif'&StartAt=0&NbRecs=10) | *H.vulgare* | CGTCA | Cis-acting regulatory element involved in the MeJA-responsiveness |
|  | [Circadian](http://bioinformatics.psb.ugent.be/webtools/plantcare/cgi-bin/show_site_info.htpl?QWhere=ID_of_Site like 'LE~circadian'&StartAt=0&NbRecs=10) | *Lycopersicon esculentum* | CAAAGATATC | Cis-acting regulatory element involved in circadian control |
|  | [CGTCA-motif](http://bioinformatics.psb.ugent.be/webtools/plantcare/cgi-bin/show_site_info.htpl?QWhere=ID_of_Site like 'HV~CGTCA-motif'&StartAt=0&NbRecs=10) | *H.vulgare* | CGTCA | Cis-acting regulatory element involved in the MeJA-responsiveness |
|  | [LTR](http://bioinformatics.psb.ugent.be/webtools/plantcare/cgi-bin/show_site_info.htpl?QWhere=ID_of_Site like 'HV~LTR'&StartAt=0&NbRecs=10) | *H. vulgare* | CCGAAA | Cis-acting element involved in low-temperature responsiveness |
|  | [MBS](http://bioinformatics.psb.ugent.be/webtools/plantcare/cgi-bin/show_site_info.htpl?QWhere=ID_of_Site like 'AT~MBS'&StartAt=0&NbRecs=10) | *A. thaliana* | CAACTG | MYB binding site involved in drought-inducibility |
|  | [TC-rich repeats](http://bioinformatics.psb.ugent.be/webtools/plantcare/cgi-bin/show_site_info.htpl?QWhere=ID_of_Site like 'NT~TC-rich repeats'&StartAt=0&NbRecs=10) | *N. tabacum* | ATTTTCTCCA | Cis-acting element involved in defense and stress responsiveness |
|  | [TGA-element](http://bioinformatics.psb.ugent.be/webtools/plantcare/cgi-bin/show_site_info.htpl?QWhere=ID_of_Site like 'BO~TGA-element'&StartAt=0&NbRecs=10) | *B. oleracea* | AACGAC | Auxin-responsive element |
|  | [TATC-box](http://bioinformatics.psb.ugent.be/webtools/plantcare/cgi-bin/show_site_info.htpl?QWhere=ID_of_Site like 'OS~TATC-box'&StartAt=0&NbRecs=10) | *Oryza sativa* | TATCCCA | Cis-acting element involved in gibberellin-responsiveness |
| BrPDI1-3 | [ABRE](http://bioinformatics.psb.ugent.be/webtools/plantcare/cgi-bin/show_site_info.htpl?QWhere=ID_of_Site like 'AT~ABRE'&StartAt=0&NbRecs=10) | A. thaliana | TACGTG | Cis-acting element involved in the abscisic acid responsiveness |
|  | [Box-W1](http://bioinformatics.psb.ugent.be/webtools/plantcare/cgi-bin/show_site_info.htpl?QWhere=ID_of_Site like 'PC~Box-W1'&StartAt=0&NbRecs=10) | *P. crispum* | TTGACC | Fungal elicitor responsive element |
|  | [CE3](http://bioinformatics.psb.ugent.be/webtools/plantcare/cgi-bin/show_site_info.htpl?QWhere=ID_of_Site like 'OS~CE3'&StartAt=0&NbRecs=10) | *O. sativa* | GACGCGTGTC | Cis-acting element involved in ABA and VP1 responsiveness |
|  | [ERE](http://bioinformatics.psb.ugent.be/webtools/plantcare/cgi-bin/show_site_info.htpl?QWhere=ID_of_Site like 'DC~ERE'&StartAt=0&NbRecs=10) | *Dianthus caryophyllu* | ATTTCAAA | Ethylene-responsive element |
|  | HSE | *B. oleracea* | AGAAAATTCG | Cis-acting element involved in heat stress responsiveness |
|  | [LTR](http://bioinformatics.psb.ugent.be/webtools/plantcare/cgi-bin/show_site_info.htpl?QWhere=ID_of_Site like 'HV~LTR'&StartAt=0&NbRecs=10) | *H. vulgare* | CCGAAA | Cis-acting element involved in low-temperature responsiveness |
|  | [MBS](http://bioinformatics.psb.ugent.be/webtools/plantcare/cgi-bin/show_site_info.htpl?QWhere=ID_of_Site like 'AT~MBS'&StartAt=0&NbRecs=10) | *A. thaliana* | CAACTG | MYB binding site involved in drought-inducibility |
|  | [TC-rich repeats](http://bioinformatics.psb.ugent.be/webtools/plantcare/cgi-bin/show_site_info.htpl?QWhere=ID_of_Site like 'NT~TC-rich repeats'&StartAt=0&NbRecs=10) | *N. tabacum* | ATTTTCTCCA | Cis-acting element involved in defense and stress responsiveness |
|  | [TCA-element](http://bioinformatics.psb.ugent.be/webtools/plantcare/cgi-bin/show_site_info.htpl?QWhere=ID_of_Site like 'BO~TCA-element'&StartAt=0&NbRecs=10) | *B. oleracea* | CAGAAAAGGA | Cis-acting element involved in salicylic acid responsiveness |
|  | [TGA-element](http://bioinformatics.psb.ugent.be/webtools/plantcare/cgi-bin/show_site_info.htpl?QWhere=ID_of_Site like 'BO~TGA-element'&StartAt=0&NbRecs=10) | *B. oleracea* | AACGAC | Auxin-responsive element |
|  | WBOX | *A. thaliana* | TTGACC | Functions in response to environmental stresses/ regulated plant defense response |
| BrPDI1-4 | [ACE](http://bioinformatics.psb.ugent.be/webtools/plantcare/cgi-bin/show_site_info.htpl?QWhere=ID_of_Site like 'PH~ACE'&StartAt=0&NbRecs=10) | *P. hortense* | ACGTGGA | Cis-acting element involved in light responsiveness |
|  | [Box-W1](http://bioinformatics.psb.ugent.be/webtools/plantcare/cgi-bin/show_site_info.htpl?QWhere=ID_of_Site like 'PC~Box-W1'&StartAt=0&NbRecs=10) | *P. crispum* | TTGACC | Fungal elicitor responsive element |
|  | [CGTCA-motif](http://bioinformatics.psb.ugent.be/webtools/plantcare/cgi-bin/show_site_info.htpl?QWhere=ID_of_Site like 'HV~CGTCA-motif'&StartAt=0&NbRecs=10) | *H.vulgare* | CGTCA | Cis-acting regulatory element involved in the MeJA-responsiveness |
|  | [I-box](http://bioinformatics.psb.ugent.be/webtools/plantcare/cgi-bin/show_site_info.htpl?QWhere=ID_of_Site like 'ZM~I-box'&StartAt=0&NbRecs=10) | *Zea mays* | GATAGGG | Part of a light responsive element |
|  | [LTR](http://bioinformatics.psb.ugent.be/webtools/plantcare/cgi-bin/show_site_info.htpl?QWhere=ID_of_Site like 'HV~LTR'&StartAt=0&NbRecs=10) | *H. vulgare* | CCGAAA | Cis-acting element involved in low-temperature responsiveness |
|  | [TC-rich repeats](http://bioinformatics.psb.ugent.be/webtools/plantcare/cgi-bin/show_site_info.htpl?QWhere=ID_of_Site like 'NT~TC-rich repeats'&StartAt=0&NbRecs=10) | *N. tabacum* | ATTTTCTCCA | Cis-acting element involved in defense and stress responsiveness |
|  | [TCA-element](http://bioinformatics.psb.ugent.be/webtools/plantcare/cgi-bin/show_site_info.htpl?QWhere=ID_of_Site like 'BO~TCA-element'&StartAt=0&NbRecs=10) | *B. oleracea* | CAGAAAAGGA | Cis-acting element involved in salicylic acid responsiveness |
|  | WBOX | *A. thaliana* | TTGACC | Functions in response to environmental stresses/ regulated plant defense response |
| BrPDI1-5 | [ABRE](http://bioinformatics.psb.ugent.be/webtools/plantcare/cgi-bin/show_site_info.htpl?QWhere=ID_of_Site like 'AT~ABRE'&StartAt=0&NbRecs=10) | A. thaliana | TACGTG | Cis-acting element involved in the abscisic acid responsiveness |
|  | [Box-W1](http://bioinformatics.psb.ugent.be/webtools/plantcare/cgi-bin/show_site_info.htpl?QWhere=ID_of_Site like 'PC~Box-W1'&StartAt=0&NbRecs=10) | *P. crispum* | TTGACC | Fungal elicitor responsive element |
|  | [CE3](http://bioinformatics.psb.ugent.be/webtools/plantcare/cgi-bin/show_site_info.htpl?QWhere=ID_of_Site like 'OS~CE3'&StartAt=0&NbRecs=10) | *O. sativa* | GACGCGTGTC | Cis-acting element involved in ABA and VP1 responsiveness |
|  | [ERE](http://bioinformatics.psb.ugent.be/webtools/plantcare/cgi-bin/show_site_info.htpl?QWhere=ID_of_Site like 'DC~ERE'&StartAt=0&NbRecs=10) | *D.caryophyllus* | ATTTCAAA | Ethylene-responsive element |
|  | HSE | *B. oleracea* | AGAAAATTCG | *Cis*-acting element involved in heat stress responsiveness |
|  | [I-box](http://bioinformatics.psb.ugent.be/webtools/plantcare/cgi-bin/show_site_info.htpl?QWhere=ID_of_Site like 'ZM~I-box'&StartAt=0&NbRecs=10) | *Z. mays* | GATAGGG | Part of a light responsive element |
|  | [MBS](http://bioinformatics.psb.ugent.be/webtools/plantcare/cgi-bin/show_site_info.htpl?QWhere=ID_of_Site like 'AT~MBS'&StartAt=0&NbRecs=10) | *A. thaliana* | CAACTG | MYB binding site involved in drought-inducibility |
|  | [TC-rich repeats](http://bioinformatics.psb.ugent.be/webtools/plantcare/cgi-bin/show_site_info.htpl?QWhere=ID_of_Site like 'NT~TC-rich repeats'&StartAt=0&NbRecs=10) | *N. tabacum* | ATTTTCTCCA | Cis-acting element involved in defense and stress responsiveness |
|  | [TCA-element](http://bioinformatics.psb.ugent.be/webtools/plantcare/cgi-bin/show_site_info.htpl?QWhere=ID_of_Site like 'BO~TCA-element'&StartAt=0&NbRecs=10) | *B. oleracea* | CAGAAAAGGA | Cis-acting element involved in salicylic acid responsiveness |
|  | [TGA-element](http://bioinformatics.psb.ugent.be/webtools/plantcare/cgi-bin/show_site_info.htpl?QWhere=ID_of_Site like 'BO~TGA-element'&StartAt=0&NbRecs=10) | *B. oleracea* | AACGAC | Auxin-responsive element |
|  | WBOX | *A. thaliana* | TTGACC | Functions in response to environmental stresses/ regulated plant defense response |
| BrPDI2-1 | [ABRE](http://bioinformatics.psb.ugent.be/webtools/plantcare/cgi-bin/show_site_info.htpl?QWhere=ID_of_Site like 'AT~ABRE'&StartAt=0&NbRecs=10) | A. thaliana | TACGTG | Cis-acting element involved in the abscisic acid responsiveness |
|  | [Box-W1](http://bioinformatics.psb.ugent.be/webtools/plantcare/cgi-bin/show_site_info.htpl?QWhere=ID_of_Site like 'PC~Box-W1'&StartAt=0&NbRecs=10) | *P. crispum* | TTGACC | Fungal elicitor responsive element |
|  | [ERE](http://bioinformatics.psb.ugent.be/webtools/plantcare/cgi-bin/show_site_info.htpl?QWhere=ID_of_Site like 'DC~ERE'&StartAt=0&NbRecs=10) | *D. caryophyllus* | ATTTCAAA | Ethylene-responsive element |
|  | HSE | *B. oleracea* | AGAAAATTCG | *Cis*-acting element involved in heat stress responsiveness |
|  | [I-box](http://bioinformatics.psb.ugent.be/webtools/plantcare/cgi-bin/show_site_info.htpl?QWhere=ID_of_Site like 'ZM~I-box'&StartAt=0&NbRecs=10) | *Z. mays* | GATAGGG | Part of a light responsive element |
|  | [LTR](http://bioinformatics.psb.ugent.be/webtools/plantcare/cgi-bin/show_site_info.htpl?QWhere=ID_of_Site like 'HV~LTR'&StartAt=0&NbRecs=10) | *H. vulgare* | CCGAAA | Cis-acting element involved in low-temperature responsiveness |
|  | WBOX | *A. thaliana* | TTGACC | Functions in response to environmental stresses/ regulated plant defense response |
| BrPDI2-2 | [ABRE](http://bioinformatics.psb.ugent.be/webtools/plantcare/cgi-bin/show_site_info.htpl?QWhere=ID_of_Site like 'AT~ABRE'&StartAt=0&NbRecs=10) | A. thaliana | TACGTG | Cis-acting element involved in the abscisic acid responsiveness |
|  | [Box-W1](http://bioinformatics.psb.ugent.be/webtools/plantcare/cgi-bin/show_site_info.htpl?QWhere=ID_of_Site like 'PC~Box-W1'&StartAt=0&NbRecs=10) | *P. crispum* | TTGACC | Fungal elicitor responsive element |
|  | [circadian](http://bioinformatics.psb.ugent.be/webtools/plantcare/cgi-bin/show_site_info.htpl?QWhere=ID_of_Site like 'LE~circadian'&StartAt=0&NbRecs=10) | *L. esculentum* | CAANNNNATC | Cis-acting regulatory element involved in circadian control |
|  | [ERE](http://bioinformatics.psb.ugent.be/webtools/plantcare/cgi-bin/show_site_info.htpl?QWhere=ID_of_Site like 'DC~ERE'&StartAt=0&NbRecs=10) | *D.caryophyllus* | ATTTCAAA | Ethylene-responsive element |
|  | [I-box](http://bioinformatics.psb.ugent.be/webtools/plantcare/cgi-bin/show_site_info.htpl?QWhere=ID_of_Site like 'ZM~I-box'&StartAt=0&NbRecs=10) | *Z. mays* | GATAGGG | Part of a light responsive element |
|  | [LTR](http://bioinformatics.psb.ugent.be/webtools/plantcare/cgi-bin/show_site_info.htpl?QWhere=ID_of_Site like 'HV~LTR'&StartAt=0&NbRecs=10) | *H. vulgare* | CCGAAA | Cis-acting element involved in low-temperature responsiveness |
|  | HSE | *B. oleracea* | AGAAAATTCG | *Cis*-acting element involved in heat stress responsiveness |
|  | [MBS](http://bioinformatics.psb.ugent.be/webtools/plantcare/cgi-bin/show_site_info.htpl?QWhere=ID_of_Site like 'AT~MBS'&StartAt=0&NbRecs=10) | *A. thaliana* | CAACTG | MYB binding site involved in drought-inducibility |
|  | [TC-rich repeats](http://bioinformatics.psb.ugent.be/webtools/plantcare/cgi-bin/show_site_info.htpl?QWhere=ID_of_Site like 'NT~TC-rich repeats'&StartAt=0&NbRecs=10) | *N. tabacum* | ATTTTCTTCA | Cis-acting element involved in defense and stress responsiveness |
|  | [TCA-element](http://bioinformatics.psb.ugent.be/webtools/plantcare/cgi-bin/show_site_info.htpl?QWhere=ID_of_Site like 'BO~TCA-element'&StartAt=0&NbRecs=10) | *B. oleracea* | CAGAAAAGGA | Cis-acting element involved in salicylic acid responsiveness |
|  | [TGA-element](http://bioinformatics.psb.ugent.be/webtools/plantcare/cgi-bin/show_site_info.htpl?QWhere=ID_of_Site like 'BO~TGA-element'&StartAt=0&NbRecs=10) | *B. oleracea* | AACGAC | Auxin-responsive element |
|  | WBOX | *A. thaliana* | TTGACC | Functions in response to environmental stresses/ regulated plant defense response |
| BrPDI2-3 | [Box-W1](http://bioinformatics.psb.ugent.be/webtools/plantcare/cgi-bin/show_site_info.htpl?QWhere=ID_of_Site like 'PC~Box-W1'&StartAt=0&NbRecs=10) | *P. crispum* | TTGACC | Fungal elicitor responsive element |
|  | [Circadian](http://bioinformatics.psb.ugent.be/webtools/plantcare/cgi-bin/show_site_info.htpl?QWhere=ID_of_Site like 'LE~circadian'&StartAt=0&NbRecs=10) | *L. esculentum* | CAANNNNATC | Cis-acting regulatory element involved in circadian control |
|  | [I-box](http://bioinformatics.psb.ugent.be/webtools/plantcare/cgi-bin/show_site_info.htpl?QWhere=ID_of_Site like 'ZM~I-box'&StartAt=0&NbRecs=10) | *Z. mays* | GATAGGG | Part of a light responsive element |
|  | [MBS](http://bioinformatics.psb.ugent.be/webtools/plantcare/cgi-bin/show_site_info.htpl?QWhere=ID_of_Site like 'AT~MBS'&StartAt=0&NbRecs=10) | *A. thaliana* | CAACTG | MYB binding site involved in drought-inducibility |
|  | [TC-rich repeats](http://bioinformatics.psb.ugent.be/webtools/plantcare/cgi-bin/show_site_info.htpl?QWhere=ID_of_Site like 'NT~TC-rich repeats'&StartAt=0&NbRecs=10) | *N. tabacum* | ATTTTCTTCA | Cis-acting element involved in defense and stress responsiveness |
|  | [TCA-element](http://bioinformatics.psb.ugent.be/webtools/plantcare/cgi-bin/show_site_info.htpl?QWhere=ID_of_Site like 'BO~TCA-element'&StartAt=0&NbRecs=10) | *B. oleracea* | CAGAAAAGGA | Cis-acting element involved in salicylic acid responsiveness |
|  | [TGA-element](http://bioinformatics.psb.ugent.be/webtools/plantcare/cgi-bin/show_site_info.htpl?QWhere=ID_of_Site like 'BO~TGA-element'&StartAt=0&NbRecs=10) | *B. oleracea* | AACGAC | Auxin-responsive element |
|  | WBOX | *A. thaliana* | TTGACC | Functions in response to environmental stresses/ regulated plant defense response |
| BrPDI3-1 | [Box-W1](http://bioinformatics.psb.ugent.be/webtools/plantcare/cgi-bin/show_site_info.htpl?QWhere=ID_of_Site like 'PC~Box-W1'&StartAt=0&NbRecs=10) | *P. crispum* | TTGACC | Fungal elicitor responsive element |
|  | [Circadian](http://bioinformatics.psb.ugent.be/webtools/plantcare/cgi-bin/show_site_info.htpl?QWhere=ID_of_Site like 'LE~circadian'&StartAt=0&NbRecs=10) | *L. esculentum* | CAANNNNATC | Cis-acting regulatory element involved in circadian control |
|  | [CE3](http://bioinformatics.psb.ugent.be/webtools/plantcare/cgi-bin/show_site_info.htpl?QWhere=ID_of_Site like 'OS~CE3'&StartAt=0&NbRecs=10) | *O. sativa* | GACGCGTGTC | Cis-acting element involved in ABA and VP1 responsiveness |
|  | [GARE-motif](http://bioinformatics.psb.ugent.be/webtools/plantcare/cgi-bin/show_site_info.htpl?QWhere=ID_of_Site like 'BO~GARE-motif'&StartAt=0&NbRecs=10) | *B. oleracea* | AAACAGA | Gibberellin-responsive element |
|  | HSE | *B. oleracea* | AGAAAATTCG | *Cis*-acting element involved in heat stress responsiveness |
|  | [LTR](http://bioinformatics.psb.ugent.be/webtools/plantcare/cgi-bin/show_site_info.htpl?QWhere=ID_of_Site like 'HV~LTR'&StartAt=0&NbRecs=10) | *H. vulgare* | CCGAAA | Cis-acting element involved in low-temperature responsiveness |
|  | [MBS](http://bioinformatics.psb.ugent.be/webtools/plantcare/cgi-bin/show_site_info.htpl?QWhere=ID_of_Site like 'AT~MBS'&StartAt=0&NbRecs=10) | *A. thaliana* | CAACTG | MYB binding site involved in drought-inducibility |
|  | [TCA-element](http://bioinformatics.psb.ugent.be/webtools/plantcare/cgi-bin/show_site_info.htpl?QWhere=ID_of_Site like 'BO~TCA-element'&StartAt=0&NbRecs=10) | *B. oleracea* | CAGAAAAGGA | Cis-acting element involved in salicylic acid responsiveness |
|  | [TGA-element](http://bioinformatics.psb.ugent.be/webtools/plantcare/cgi-bin/show_site_info.htpl?QWhere=ID_of_Site like 'BO~TGA-element'&StartAt=0&NbRecs=10) | *B. oleracea* | AACGAC | Auxin-responsive element |
|  | WBOX | *A. thaliana* | TTGACC | Functions in response to environmental stresses/ regulated plant defense response |
| BrPDI3-2 | [ABRE](http://bioinformatics.psb.ugent.be/webtools/plantcare/cgi-bin/show_site_info.htpl?QWhere=ID_of_Site like 'AT~ABRE'&StartAt=0&NbRecs=10) | A. thaliana | TACGTG | Cis-acting element involved in the abscisic acid responsiveness |
|  | [Box-W1](http://bioinformatics.psb.ugent.be/webtools/plantcare/cgi-bin/show_site_info.htpl?QWhere=ID_of_Site like 'PC~Box-W1'&StartAt=0&NbRecs=10) | *P.crispum* | TTGACC | Fungal elicitor responsive element |
|  | [Circadian](http://bioinformatics.psb.ugent.be/webtools/plantcare/cgi-bin/show_site_info.htpl?QWhere=ID_of_Site like 'LE~circadian'&StartAt=0&NbRecs=10) | *L. esculentum* | CAANNNNATC | Cis-acting regulatory element involved in circadian control |
|  | [GARE-motif](http://bioinformatics.psb.ugent.be/webtools/plantcare/cgi-bin/show_site_info.htpl?QWhere=ID_of_Site like 'BO~GARE-motif'&StartAt=0&NbRecs=10) | *B. oleracea* | AAACAGA | Gibberellin-responsive element |
|  | [GAG-motif](http://bioinformatics.psb.ugent.be/webtools/plantcare/cgi-bin/show_site_info.htpl?QWhere=ID_of_Site like 'HV~GAG-motif'&StartAt=0&NbRecs=10) | *H. vulgare* | GGAGATG | Part of a light responsive element |
|  | HSE | *B. oleracea* | AGAAAATTCG | *Cis*-acting element involved in heat stress responsiveness |
|  | [LTR](http://bioinformatics.psb.ugent.be/webtools/plantcare/cgi-bin/show_site_info.htpl?QWhere=ID_of_Site like 'HV~LTR'&StartAt=0&NbRecs=10) | *H. vulgare* | CCGAAA | Cis-acting element involved in low-temperature responsiveness |
|  | [MBS](http://bioinformatics.psb.ugent.be/webtools/plantcare/cgi-bin/show_site_info.htpl?QWhere=ID_of_Site like 'AT~MBS'&StartAt=0&NbRecs=10) | *A. thaliana* | CAACTG | MYB binding site involved in drought-inducibility |
|  | [TGA-element](http://bioinformatics.psb.ugent.be/webtools/plantcare/cgi-bin/show_site_info.htpl?QWhere=ID_of_Site like 'BO~TGA-element'&StartAt=0&NbRecs=10) | *B. oleracea* | AACGAC | Auxin-responsive element |
|  | [TCA-element](http://bioinformatics.psb.ugent.be/webtools/plantcare/cgi-bin/show_site_info.htpl?QWhere=ID_of_Site like 'BO~TCA-element'&StartAt=0&NbRecs=10) | *B. oleracea* | CAGAAAAGGA | Cis-acting element involved in salicylic acid responsiveness |
|  | WBOX | *A. thaliana* | TTGACC | Functions in response to environmental stresses/ regulated plant defense response |
| BrPDI4-1 | [ABRE](http://bioinformatics.psb.ugent.be/webtools/plantcare/cgi-bin/show_site_info.htpl?QWhere=ID_of_Site like 'AT~ABRE'&StartAt=0&NbRecs=10) | A. thaliana | TACGTG | Cis-acting element involved in the abscisic acid responsiveness |
|  | [AE-box](http://bioinformatics.psb.ugent.be/webtools/plantcare/cgi-bin/show_site_info.htpl?QWhere=ID_of_Site like 'AT~AE-box'&StartAt=0&NbRecs=10) | *A. thaliana* | AGAAACTT | Part of a module for light response |
|  | [Box-W1](http://bioinformatics.psb.ugent.be/webtools/plantcare/cgi-bin/show_site_info.htpl?QWhere=ID_of_Site like 'PC~Box-W1'&StartAt=0&NbRecs=10) | *P. crispum* | TTGACC | Fungal elicitor responsive element |
|  | [Circadian](http://bioinformatics.psb.ugent.be/webtools/plantcare/cgi-bin/show_site_info.htpl?QWhere=ID_of_Site like 'LE~circadian'&StartAt=0&NbRecs=10) | *L. esculentum* | CAANNNNATC | Cis-acting regulatory element involved in circadian control |
|  | [CE3](http://bioinformatics.psb.ugent.be/webtools/plantcare/cgi-bin/show_site_info.htpl?QWhere=ID_of_Site like 'OS~CE3'&StartAt=0&NbRecs=10) | *O. sativa* | GACGCGTGTC | Cis-acting element involved in ABA and VP1 responsiveness |
|  | [LTR](http://bioinformatics.psb.ugent.be/webtools/plantcare/cgi-bin/show_site_info.htpl?QWhere=ID_of_Site like 'HV~LTR'&StartAt=0&NbRecs=10) | *H. vulgare* | CCGAAA | Cis-acting element involved in low-temperature responsiveness |
|  | [CAT-box](http://bioinformatics.psb.ugent.be/webtools/plantcare/cgi-bin/show_site_info.htpl?QWhere=ID_of_Site like 'AT~CAT-box'&StartAt=0&NbRecs=10) | *A. thaliana* | GCCACT | Cis-acting regulatory element related to meristem expression |
|  | [MBSI](http://bioinformatics.psb.ugent.be/webtools/plantcare/cgi-bin/show_site_info.htpl?QWhere=ID_of_Site like 'PH~MBSI'&StartAt=0&NbRecs=10) | *P. hybrida* | AAAAAAC(G/C)GTTA | MYB binding site involved in flavonoid biosynthetic genes regulation |
|  | [TCA-element](http://bioinformatics.psb.ugent.be/webtools/plantcare/cgi-bin/show_site_info.htpl?QWhere=ID_of_Site like 'BO~TCA-element'&StartAt=0&NbRecs=10) | *B. oleracea* | CAGAAAAGGA | Cis-acting element involved in salicylic acid responsiveness |
|  | [TGA-element](http://bioinformatics.psb.ugent.be/webtools/plantcare/cgi-bin/show_site_info.htpl?QWhere=ID_of_Site like 'BO~TGA-element'&StartAt=0&NbRecs=10) | *B. oleracea* | AACGAC | Auxin-responsive element |
|  | WBOX | *A. thaliana* | TTGACC | Functions in response to environmental stresses/ regulated plant defense response |
| BrPDI4-2 | [Box-W1](http://bioinformatics.psb.ugent.be/webtools/plantcare/cgi-bin/show_site_info.htpl?QWhere=ID_of_Site like 'PC~Box-W1'&StartAt=0&NbRecs=10) | *P.crispum* | TTGACC | Fungal elicitor responsive element |
|  | [Circadian](http://bioinformatics.psb.ugent.be/webtools/plantcare/cgi-bin/show_site_info.htpl?QWhere=ID_of_Site like 'LE~circadian'&StartAt=0&NbRecs=10) | *L. esculentum* | CAANNNNATC | Cis-acting regulatory element involved in circadian control |
|  | [CAT-box](http://bioinformatics.psb.ugent.be/webtools/plantcare/cgi-bin/show_site_info.htpl?QWhere=ID_of_Site like 'AT~CAT-box'&StartAt=0&NbRecs=10) | *A.thaliana* | GCCACT | Cis-acting regulatory element related to meristem expression |
|  | [CE3](http://bioinformatics.psb.ugent.be/webtools/plantcare/cgi-bin/show_site_info.htpl?QWhere=ID_of_Site like 'OS~CE3'&StartAt=0&NbRecs=10) | *O. sativa* | GACGCGTGTC | Cis-acting element involved in ABA and VP1 responsiveness |
|  | [LTR](http://bioinformatics.psb.ugent.be/webtools/plantcare/cgi-bin/show_site_info.htpl?QWhere=ID_of_Site like 'HV~LTR'&StartAt=0&NbRecs=10) | *H. vulgare* | CCGAAA | Cis-acting element involved in low-temperature responsiveness |
|  | [MBS](http://bioinformatics.psb.ugent.be/webtools/plantcare/cgi-bin/show_site_info.htpl?QWhere=ID_of_Site like 'AT~MBS'&StartAt=0&NbRecs=10) | *A. thaliana* | CAACTG | MYB binding site involved in drought-inducibility |
|  | [MBSI](http://bioinformatics.psb.ugent.be/webtools/plantcare/cgi-bin/show_site_info.htpl?QWhere=ID_of_Site like 'PH~MBSI'&StartAt=0&NbRecs=10) | *P. hybrida* | AAAAAAC(G/C)GTTA | MYB binding site involved in flavonoid biosynthetic genes regulation |
|  | [TC-rich repeats](http://bioinformatics.psb.ugent.be/webtools/plantcare/cgi-bin/show_site_info.htpl?QWhere=ID_of_Site like 'NT~TC-rich repeats'&StartAt=0&NbRecs=10) | *N. tabacum* | GTTTTCTTAC | Cis-acting element involved in defense and stress responsiveness |
|  | [TCA-element](http://bioinformatics.psb.ugent.be/webtools/plantcare/cgi-bin/show_site_info.htpl?QWhere=ID_of_Site like 'BO~TCA-element'&StartAt=0&NbRecs=10) | *B. oleracea* | CAGAAAAGGA | Cis-acting element involved in salicylic acid responsiveness |
|  | [TGA-element](http://bioinformatics.psb.ugent.be/webtools/plantcare/cgi-bin/show_site_info.htpl?QWhere=ID_of_Site like 'BO~TGA-element'&StartAt=0&NbRecs=10) | *B. oleracea* | AACGAC | Auxin-responsive element |
|  | WBOX | *A. thaliana* | TTGACC | Functions in response to environmental stresses/ regulated plant defense response |
| BrPDI5-1 | [ABRE](http://bioinformatics.psb.ugent.be/webtools/plantcare/cgi-bin/show_site_info.htpl?QWhere=ID_of_Site like 'AT~ABRE'&StartAt=0&NbRecs=10) | A. thaliana | TACGTG | Cis-acting element involved in the abscisic acid responsiveness |
|  | [Box-W1](http://bioinformatics.psb.ugent.be/webtools/plantcare/cgi-bin/show_site_info.htpl?QWhere=ID_of_Site like 'PC~Box-W1'&StartAt=0&NbRecs=10) | *P.crispum* | TTGACC | Fungal elicitor responsive element |
|  | [CAT-box](http://bioinformatics.psb.ugent.be/webtools/plantcare/cgi-bin/show_site_info.htpl?QWhere=ID_of_Site like 'AT~CAT-box'&StartAt=0&NbRecs=10) | *A. thaliana* | GCCACT | Cis-acting regulatory element related to meristem expression |
|  | [CE3](http://bioinformatics.psb.ugent.be/webtools/plantcare/cgi-bin/show_site_info.htpl?QWhere=ID_of_Site like 'OS~CE3'&StartAt=0&NbRecs=10) | *O. sativa* | GACGCGTGTC | Cis-acting element involved in ABA and VP1 responsiveness |
|  | [GC-motif](http://bioinformatics.psb.ugent.be/webtools/plantcare/cgi-bin/show_site_info.htpl?QWhere=ID_of_Site like 'ZM~GC-motif'&StartAt=0&NbRecs=10) | *Z. mays* | CCCCCG | Enhancer-like element involved in anoxic specific inducibility |
|  | [MBS](http://bioinformatics.psb.ugent.be/webtools/plantcare/cgi-bin/show_site_info.htpl?QWhere=ID_of_Site like 'AT~MBS'&StartAt=0&NbRecs=10) | *A. thaliana* | CAACTG | MYB binding site involved in drought-inducibility |
|  | [TC-rich repeats](http://bioinformatics.psb.ugent.be/webtools/plantcare/cgi-bin/show_site_info.htpl?QWhere=ID_of_Site like 'NT~TC-rich repeats'&StartAt=0&NbRecs=10) | *N. tabacum* | ATTTTCTTCA | Cis-acting element involved in defense and stress responsiveness |
|  | [TGA-element](http://bioinformatics.psb.ugent.be/webtools/plantcare/cgi-bin/show_site_info.htpl?QWhere=ID_of_Site like 'BO~TGA-element'&StartAt=0&NbRecs=10) | *B. oleracea* | AACGAC | Auxin-responsive element |
|  | WBOX | *A. thaliana* | TTGACC | Functions in response to environmental stresses/ regulated plant defense response |
| BrPDI5-2 | [ABRE](http://bioinformatics.psb.ugent.be/webtools/plantcare/cgi-bin/show_site_info.htpl?QWhere=ID_of_Site like 'AT~ABRE'&StartAt=0&NbRecs=10) | A. thaliana | TACGTG | Cis-acting element involved in the abscisic acid responsiveness |
|  | [Box-W1](http://bioinformatics.psb.ugent.be/webtools/plantcare/cgi-bin/show_site_info.htpl?QWhere=ID_of_Site like 'PC~Box-W1'&StartAt=0&NbRecs=10) | *P.crispum* | TTGACC | Fungal elicitor responsive element |
|  | [Circadian](http://bioinformatics.psb.ugent.be/webtools/plantcare/cgi-bin/show_site_info.htpl?QWhere=ID_of_Site like 'LE~circadian'&StartAt=0&NbRecs=10) | *L. esculentum* | CAANNNNATC | Cis-acting regulatory element involved in circadian control |
|  | [CE3](http://bioinformatics.psb.ugent.be/webtools/plantcare/cgi-bin/show_site_info.htpl?QWhere=ID_of_Site like 'OS~CE3'&StartAt=0&NbRecs=10) | *O. sativa* | GACGCGTGTC | Cis-acting element involved in ABA and VP1 responsiveness |
|  | [GC-motif](http://bioinformatics.psb.ugent.be/webtools/plantcare/cgi-bin/show_site_info.htpl?QWhere=ID_of_Site like 'ZM~GC-motif'&StartAt=0&NbRecs=10) | *Z. mays* | CCCCCG | Enhancer-like element involved in anoxic specific inducibility |
|  | [LTR](http://bioinformatics.psb.ugent.be/webtools/plantcare/cgi-bin/show_site_info.htpl?QWhere=ID_of_Site like 'HV~LTR'&StartAt=0&NbRecs=10) | *H. vulgare* | CCGAAA | Cis-acting element involved in low-temperature responsiveness |
|  | HSE | *B. oleracea* | AGAAAATTCG | *Cis*-acting element involved in heat stress responsiveness |
|  | [MBS](http://bioinformatics.psb.ugent.be/webtools/plantcare/cgi-bin/show_site_info.htpl?QWhere=ID_of_Site like 'AT~MBS'&StartAt=0&NbRecs=10) | *A. thaliana* | CAACTG | MYB binding site involved in drought-inducibility |
|  | [TC-rich repeats](http://bioinformatics.psb.ugent.be/webtools/plantcare/cgi-bin/show_site_info.htpl?QWhere=ID_of_Site like 'NT~TC-rich repeats'&StartAt=0&NbRecs=10) | *N. tabacum* | ATTTTCTTCA | Cis-acting element involved in defense and stress responsiveness |
|  | [TGA-element](http://bioinformatics.psb.ugent.be/webtools/plantcare/cgi-bin/show_site_info.htpl?QWhere=ID_of_Site like 'BO~TGA-element'&StartAt=0&NbRecs=10) | *B. oleracea* | AACGAC | Auxin-responsive element |
| BrPDI6-1 | [ABRE](http://bioinformatics.psb.ugent.be/webtools/plantcare/cgi-bin/show_site_info.htpl?QWhere=ID_of_Site like 'AT~ABRE'&StartAt=0&NbRecs=10) | A. thaliana | TACGTG | Cis-acting element involved in the abscisic acid responsiveness |
|  | [Circadian](http://bioinformatics.psb.ugent.be/webtools/plantcare/cgi-bin/show_site_info.htpl?QWhere=ID_of_Site like 'LE~circadian'&StartAt=0&NbRecs=10) | L. esculentum | CAANNNNATC | Cis-acting regulatory element involved in circadian control |
|  | HSE | *B. oleracea* | AGAAAATTCG | Cis-acting element involved in heat stress responsiveness |
|  | [LTR](http://bioinformatics.psb.ugent.be/webtools/plantcare/cgi-bin/show_site_info.htpl?QWhere=ID_of_Site like 'HV~LTR'&StartAt=0&NbRecs=10) | *H. vulgare* | CCGAAA | Cis-acting element involved in low-temperature responsiveness |
|  | [MBS](http://bioinformatics.psb.ugent.be/webtools/plantcare/cgi-bin/show_site_info.htpl?QWhere=ID_of_Site like 'AT~MBS'&StartAt=0&NbRecs=10) | *A. thaliana* | CAACTG | MYB binding site involved in drought-inducibility |
|  | [P-box](http://bioinformatics.psb.ugent.be/webtools/plantcare/cgi-bin/show_site_info.htpl?QWhere=ID_of_Site like 'OS~P-box'&StartAt=0&NbRecs=10) | O. sativa | GCCTTTTGAGT | Gibberellin-responsive element |
|  | WBOX | *A. thaliana* | TTGACC | Functions in response to environmental stresses/ regulated plant defense response |
| BrPDI7-1 | [Circadian](http://bioinformatics.psb.ugent.be/webtools/plantcare/cgi-bin/show_site_info.htpl?QWhere=ID_of_Site like 'LE~circadian'&StartAt=0&NbRecs=10) | *L. esculentum* | CAANNNNATC | Cis-acting regulatory element involved in circadian control |
|  | HSE | *B. oleracea* | AGAAAATTCG | Cis-acting element involved in heat stress responsiveness |
|  | [LTR](http://bioinformatics.psb.ugent.be/webtools/plantcare/cgi-bin/show_site_info.htpl?QWhere=ID_of_Site like 'HV~LTR'&StartAt=0&NbRecs=10) | *H. vulgare* | CCGAAA | Cis-acting element involved in low-temperature responsiveness |
|  | [MBS](http://bioinformatics.psb.ugent.be/webtools/plantcare/cgi-bin/show_site_info.htpl?QWhere=ID_of_Site like 'AT~MBS'&StartAt=0&NbRecs=10) | *A. thaliana* | CAACTG | MYB binding site involved in drought-inducibility |
|  | [TCA-element](http://bioinformatics.psb.ugent.be/webtools/plantcare/cgi-bin/show_site_info.htpl?QWhere=ID_of_Site like 'BO~TCA-element'&StartAt=0&NbRecs=10) | *B. oleracea* | GAGAAGAATA | Cis-acting element involved in salicylic acid responsiveness |
|  | [TC-rich repeats](http://bioinformatics.psb.ugent.be/webtools/plantcare/cgi-bin/show_site_info.htpl?QWhere=ID_of_Site like 'NT~TC-rich repeats'&StartAt=0&NbRecs=10) | *N. tabacum* | ATTTTCTTCA | Cis-acting element involved in defense and stress responsiveness |
|  | WBOX | *A. thaliana* | TTGACC | Functions in response to environmental stresses/ regulated plant defense response |
| BrPDI8-1 | [ABRE](http://bioinformatics.psb.ugent.be/webtools/plantcare/cgi-bin/show_site_info.htpl?QWhere=ID_of_Site like 'AT~ABRE'&StartAt=0&NbRecs=10) | A. thaliana | TACGTG | Cis-acting element involved in the abscisic acid responsiveness |
|  | [CAT-box](http://bioinformatics.psb.ugent.be/webtools/plantcare/cgi-bin/show_site_info.htpl?QWhere=ID_of_Site like 'AT~CAT-box'&StartAt=0&NbRecs=10) | *A. thaliana* | GCCACT | Cis-acting regulatory element related to meristem expression |
|  | [Circadian](http://bioinformatics.psb.ugent.be/webtools/plantcare/cgi-bin/show_site_info.htpl?QWhere=ID_of_Site like 'LE~circadian'&StartAt=0&NbRecs=10) | *L. esculentum* | CAANNNNATC | Cis-acting regulatory element involved in circadian control |
|  | [LTR](http://bioinformatics.psb.ugent.be/webtools/plantcare/cgi-bin/show_site_info.htpl?QWhere=ID_of_Site like 'HV~LTR'&StartAt=0&NbRecs=10) | *H. vulgare* | CCGAAA | Cis-acting element involved in low-temperature responsiveness |
|  | HSE | *B. oleracea* | AGAAAATTCG | *Cis*-acting element involved in heat stress responsiveness |
|  | [MBS](http://bioinformatics.psb.ugent.be/webtools/plantcare/cgi-bin/show_site_info.htpl?QWhere=ID_of_Site like 'AT~MBS'&StartAt=0&NbRecs=10) | *A. thaliana* | CAACTG | MYB binding site involved in drought-inducibility |
|  | [MRE](http://bioinformatics.psb.ugent.be/webtools/plantcare/cgi-bin/show_site_info.htpl?QWhere=ID_of_Site like 'PC~MRE'&StartAt=0&NbRecs=10) | *P. crispum* | AACCTAA | MYB binding site involved in light responsiveness |
|  | [TGA-element](http://bioinformatics.psb.ugent.be/webtools/plantcare/cgi-bin/show_site_info.htpl?QWhere=ID_of_Site like 'BO~TGA-element'&StartAt=0&NbRecs=10) | *B.oleracea* | AACGAC | Auxin-responsive element |
|  | WBOX | *A. thaliana* | TTGACC | Functions in response to environmental stresses/ regulated plant defense response |
| BrPDI8-2 | [MBS](http://bioinformatics.psb.ugent.be/webtools/plantcare/cgi-bin/show_site_info.htpl?QWhere=ID_of_Site like 'AT~MBS'&StartAt=0&NbRecs=10) | *A. thaliana* | CAACTG | MYB binding site involved in drought-inducibility |
|  | [CE3](http://bioinformatics.psb.ugent.be/webtools/plantcare/cgi-bin/show_site_info.htpl?QWhere=ID_of_Site like 'OS~CE3'&StartAt=0&NbRecs=10) | *O. sativa* | GACGCGTGTC | Cis-acting element involved in ABA and VP1 responsiveness |
|  | [TCA-element](http://bioinformatics.psb.ugent.be/webtools/plantcare/cgi-bin/show_site_info.htpl?QWhere=ID_of_Site like 'BO~TCA-element'&StartAt=0&NbRecs=10) | *B.oleracea* | GAGAAGAATA | Cis-acting element involved in salicylic acid responsiveness |
|  | [TC-rich repeats](http://bioinformatics.psb.ugent.be/webtools/plantcare/cgi-bin/show_site_info.htpl?QWhere=ID_of_Site like 'NT~TC-rich repeats'&StartAt=0&NbRecs=10) | *N.tabacum* | ATTTTCTTCA | Cis-acting element involved in defense and stress responsiveness |
|  | [TGA-element](http://bioinformatics.psb.ugent.be/webtools/plantcare/cgi-bin/show_site_info.htpl?QWhere=ID_of_Site like 'BO~TGA-element'&StartAt=0&NbRecs=10) | *B.oleracea* | AACGAC | Auxin-responsive element |
|  | [5UTR Py-rich stretch](http://bioinformatics.psb.ugent.be/webtools/plantcare/cgi-bin/show_site_info.htpl?QWhere=ID_of_Site like 'LE~5UTR Py-rich stretch'&StartAt=0&NbRecs=10) | *L. esculentum* | TTTCTTCTCT | Cis-acting element conferring high transcription levels |
| BrPDI8-3 | [ABRE](http://bioinformatics.psb.ugent.be/webtools/plantcare/cgi-bin/show_site_info.htpl?QWhere=ID_of_Site like 'AT~ABRE'&StartAt=0&NbRecs=10) | A. thaliana | TACGTG | Cis-acting element involved in the abscisic acid responsiveness |
|  | [CE3](http://bioinformatics.psb.ugent.be/webtools/plantcare/cgi-bin/show_site_info.htpl?QWhere=ID_of_Site like 'OS~CE3'&StartAt=0&NbRecs=10) | *O. sativa* | GACGCGTGTC | Cis-acting element involved in ABA and VP1 responsiveness |
|  | [LTR](http://bioinformatics.psb.ugent.be/webtools/plantcare/cgi-bin/show_site_info.htpl?QWhere=ID_of_Site like 'HV~LTR'&StartAt=0&NbRecs=10) | *H. vulgare* | CCGAAA | Cis-acting element involved in low-temperature responsiveness |
|  | [Circadian](http://bioinformatics.psb.ugent.be/webtools/plantcare/cgi-bin/show_site_info.htpl?QWhere=ID_of_Site like 'LE~circadian'&StartAt=0&NbRecs=10) | *L. esculentum* | CAANNNNATC | Cis-acting regulatory element involved in circadian control |
|  | [MBS](http://bioinformatics.psb.ugent.be/webtools/plantcare/cgi-bin/show_site_info.htpl?QWhere=ID_of_Site like 'AT~MBS'&StartAt=0&NbRecs=10) | *A. thaliana* | CAACTG | MYB binding site involved in drought-inducibility |
|  | TCA-element | *B. oleracea* | GAGAAGAATA | *Cis-*acting element involved in salicylic acid responsiveness |
|  | [TC-rich repeats](http://bioinformatics.psb.ugent.be/webtools/plantcare/cgi-bin/show_site_info.htpl?QWhere=ID_of_Site like 'NT~TC-rich repeats'&StartAt=0&NbRecs=10) | *N. tabacum* | ATTTTCTTCA | Cis-acting element involved in defense and stress responsiveness |
|  | [TGA-element](http://bioinformatics.psb.ugent.be/webtools/plantcare/cgi-bin/show_site_info.htpl?QWhere=ID_of_Site like 'BO~TGA-element'&StartAt=0&NbRecs=10) | *B.oleracea* | AACGAC | Auxin-responsive element |
|  | WBOX | *A. thaliana* | TTGACC | Functions in response to environmental stresses/ regulated plant defense response |
| BrPDI8-4 | [ABRE](http://bioinformatics.psb.ugent.be/webtools/plantcare/cgi-bin/show_site_info.htpl?QWhere=ID_of_Site like 'AT~ABRE'&StartAt=0&NbRecs=10) | A. thaliana | TACGTG | Cis-acting element involved in the abscisic acid responsiveness |
|  | [Circadian](http://bioinformatics.psb.ugent.be/webtools/plantcare/cgi-bin/show_site_info.htpl?QWhere=ID_of_Site like 'LE~circadian'&StartAt=0&NbRecs=10) | *L. esculentum* | CAANNNNATC | Cis-acting regulatory element involved in circadian control |
|  | [LTR](http://bioinformatics.psb.ugent.be/webtools/plantcare/cgi-bin/show_site_info.htpl?QWhere=ID_of_Site like 'HV~LTR'&StartAt=0&NbRecs=10) | *H. vulgare* | CCGAAA | Cis-acting element involved in low-temperature responsiveness |
|  | [MBS](http://bioinformatics.psb.ugent.be/webtools/plantcare/cgi-bin/show_site_info.htpl?QWhere=ID_of_Site like 'AT~MBS'&StartAt=0&NbRecs=10) | *A. thaliana* | CAACTG | MYB binding site involved in drought-inducibility |
|  | [TCA-element](http://bioinformatics.psb.ugent.be/webtools/plantcare/cgi-bin/show_site_info.htpl?QWhere=ID_of_Site like 'BO~TCA-element'&StartAt=0&NbRecs=10) | *B. oleracea* | CAGAAAAGGA | Cis-acting element involved in salicylic acid responsiveness |
|  | [TC-rich repeats](http://bioinformatics.psb.ugent.be/webtools/plantcare/cgi-bin/show_site_info.htpl?QWhere=ID_of_Site like 'NT~TC-rich repeats'&StartAt=0&NbRecs=10) | *N. tabacum* | ATTTTCTTCA | Cis-acting element involved in defense and stress responsiveness |
|  | [TGA-element](http://bioinformatics.psb.ugent.be/webtools/plantcare/cgi-bin/show_site_info.htpl?QWhere=ID_of_Site like 'BO~TGA-element'&StartAt=0&NbRecs=10) | *B. oleracea* | AACGAC | Auxin-responsive element |
|  | WBOX | *A. thaliana* | TTGACC | Functions in response to environmental stresses/ regulated plant defense response |
|  | Circadian | *S. lycopersicum* | CAANNNNATC | *Cis* acting regulatory element involved in circadian control |
| BrPDI8-5 | ARFAT | *A. thaliana* | TGTCTC | Responsive to ABA and auxin |
|  | Circadian | *S. lycopersicum* | CAANNNNATC | *Cis* acting regulatory element involved in circadian control |
|  | [CE3](http://bioinformatics.psb.ugent.be/webtools/plantcare/cgi-bin/show_site_info.htpl?QWhere=ID_of_Site like 'OS~CE3'&StartAt=0&NbRecs=10) | *O. sativa* | GACGCGTGTC | Cis-acting element involved in ABA and VP1 responsiveness |
|  | LTR | *A. thaliana* | CCGAAA | Low-temperature-responsive |
|  | TCA-element | *B. oleracea* | GAGAAGAATA | *Cis-*acting element involved in salicylic acid responsiveness |
| BrPDI8-6 | Circadian | *S. lycopersicum* | CAANNNNATC | *Cis* acting regulatory element involved in circadian control |
|  | [CE3](http://bioinformatics.psb.ugent.be/webtools/plantcare/cgi-bin/show_site_info.htpl?QWhere=ID_of_Site like 'OS~CE3'&StartAt=0&NbRecs=10) | *O. sativa* | GACGCGTGTC | Cis-acting element involved in ABA and VP1 responsiveness |
|  | LTR | *A. thaliana* | CCGAAA | Low-temperature-responsive |
|  | HSE | *B. oleracea* | AGAAAATTCG | *Cis*-acting element involved in heat stress responsiveness |
|  | [MBS](http://bioinformatics.psb.ugent.be/webtools/plantcare/cgi-bin/show_site_info.htpl?QWhere=ID_of_Site like 'AT~MBS'&StartAt=0&NbRecs=10) | *A. thaliana* | CAACTG | MYB binding site involved in drought-inducibility |
| BrPDI9-1 | [ABRE](http://bioinformatics.psb.ugent.be/webtools/plantcare/cgi-bin/show_site_info.htpl?QWhere=ID_of_Site like 'AT~ABRE'&StartAt=0&NbRecs=10) | A. thaliana | TACGTG | Cis-acting element involved in the abscisic acid responsiveness |
|  | Circadian | *S. lycopersicum* | CAANNNNATC | *Cis* acting regulatory element involved in circadian control |
|  | [CE3](http://bioinformatics.psb.ugent.be/webtools/plantcare/cgi-bin/show_site_info.htpl?QWhere=ID_of_Site like 'OS~CE3'&StartAt=0&NbRecs=10) | *O. sativa* | GACGCGTGTC | Cis-acting element involved in ABA and VP1 responsiveness |
|  | HSE | *B. oleracea* | AGAAAATTCG | *Cis*-acting element involved in heat stress responsiveness |
|  | LTR | *A. thaliana* | CCGAAA | Low-temperature-responsive |
|  | MBS | *A. thaliana* | CAACTG | MYB binding site involved in drought-inducibility |
|  | TCA-element | *B. oleracea* | GAGAAGAATA | *Cis-*acting element involved in salicylic acid responsiveness |
|  | TC-rich repeats | *N. tabacum* | ATTTTCTTCA | *Cis-*acting element involved in defense and stress responsiveness |
|  | WBOX | *A. thaliana* | TTGAC | Functions in response to environmental stresses/ regulated plant defense response |
| BrPDI9-2 | ARFAT | *A. thaliana* | TGTCTC | Responsive to ABA and auxin |
|  | [CE3](http://bioinformatics.psb.ugent.be/webtools/plantcare/cgi-bin/show_site_info.htpl?QWhere=ID_of_Site like 'OS~CE3'&StartAt=0&NbRecs=10) | *O. sativa* | GACGCGTGTC | Cis-acting element involved in ABA and VP1 responsiveness |
|  | LTR | *A. thaliana* | CCGAAA | Low-temperature-responsive |
|  | MBS | *A. thaliana* | CAACTG | MYB binding site involved in drought-inducibility |
|  | TCA-element | *B. oleracea* | GAGAAGAATA | *Cis-*acting element involved in salicylic acid responsiveness |
|  | TC-rich repeats | *N. tabacum* | ATTTTCTTCA | *Cis-*acting element involved in defense and stress responsiveness |
| BrPDI10-1 | Circadian | *S. lycopersicum* | CAANNNNATC | *Cis* acting regulatory element involved in circadian control |
|  | HSE | *B. oleracea* | AGAAAATTCG | *Cis*-acting element involved in heat stress responsiveness |
|  | LTR | *A. thaliana* | CCGAAA | Low-temperature-responsive |
|  | [MBS](http://bioinformatics.psb.ugent.be/webtools/plantcare/cgi-bin/show_site_info.htpl?QWhere=ID_of_Site like 'AT~MBS'&StartAt=0&NbRecs=10) | *A. thaliana* | CAACTG | MYB binding site involved in drought-inducibility |
|  | TC-rich repeats | *N. tabacum* | ATTTTCTTCA | *Cis-*acting element involved in defense and stress responsiveness |
|  | TCA-element | *B. oleracea* | GAGAAGAATA | *Cis-*acting element involved in salicylic acid responsiveness |
|  | WBOX | *A. thaliana* | TTGAC | Functions in response to environmental stresses/ regulated plant defense response |
| BrPDI10-2 | Circadian | *S. lycopersicum* | CAANNNNATC | *Cis* acting regulatory element involved in circadian control |
|  | LTR | *A. thaliana* | CCGAAA | Low-temperature-responsive |
|  | [MBS](http://bioinformatics.psb.ugent.be/webtools/plantcare/cgi-bin/show_site_info.htpl?QWhere=ID_of_Site like 'AT~MBS'&StartAt=0&NbRecs=10) | *A. thaliana* | CAACTG | MYB binding site involved in drought-inducibility |
|  | TC-rich repeats | *N. tabacum* | ATTTTCTTCA | *Cis-*acting element involved in defense and stress responsiveness |
|  | TCA-element | *B. oleracea* | GAGAAGAATA | *Cis-*acting element involved in salicylic acid responsiveness |
|  | WBOX | *A. thaliana* | TTGAC | Functions in response to environmental stresses/ regulated plant defense response |
| BrPDI10-3 | [ABRE](http://bioinformatics.psb.ugent.be/webtools/plantcare/cgi-bin/show_site_info.htpl?QWhere=ID_of_Site like 'AT~ABRE'&StartAt=0&NbRecs=10) | A. thaliana | TACGTG | Cis-acting element involved in the abscisic acid responsiveness |
|  | [I-box](http://bioinformatics.psb.ugent.be/webtools/plantcare/cgi-bin/show_site_info.htpl?QWhere=ID_of_Site like 'ZM~I-box'&StartAt=0&NbRecs=10) | *Z. mays* | GATAGGG | Part of a light responsive element |
|  | Circadian | *S. lycopersicum* | CAANNNNATC | *Cis* acting regulatory element involved in circadian control |
|  | LTR | *A. thaliana* | CCGAAA | Low-temperature-responsive |
|  | TCA-element | *B. oleracea* | GAGAAGAATA | *Cis-*acting element involved in salicylic acid responsiveness |
| BrPDI10-4 | HSE | *B. oleracea* | AGAAAATTCG | *Cis*-acting element involved in heat stress responsiveness |
|  | TCA-element | *B. oleracea* | GAGAAGAATA | *Cis-*acting element involved in salicylic acid responsiveness |
|  | LTR | *A. thaliana* | CCGAAA | Low-temperature-responsive |
|  | TC-rich repeats | *N. tabacum* | ATTTTCTTCA | *Cis-*acting element involved in defense and stress responsiveness |
|  | WBOX | *A. thaliana* | TTGAC | Functions in response to environmental stresses/ regulated plant defense response |
| BrPDI11-1 | [ABRE](http://bioinformatics.psb.ugent.be/webtools/plantcare/cgi-bin/show_site_info.htpl?QWhere=ID_of_Site like 'AT~ABRE'&StartAt=0&NbRecs=10) | A. thaliana | TACGTG | Cis-acting element involved in the abscisic acid responsiveness |
|  | Circadian | *S. lycopersicum* | CAANNNNATC | *Cis* acting regulatory element involved in circadian control |
|  | LTR | *A. thaliana* | CCGAAA | Low-temperature-responsive |
|  | [MRE](http://bioinformatics.psb.ugent.be/webtools/plantcare/cgi-bin/show_site_info.htpl?QWhere=ID_of_Site like 'PC~MRE'&StartAt=0&NbRecs=10) | *P. crispum* | AACCTAA | MYB binding site involved in light responsiveness |
|  | [TC-rich repeats](http://bioinformatics.psb.ugent.be/webtools/plantcare/cgi-bin/show_site_info.htpl?QWhere=ID_of_Site like 'NT~TC-rich repeats'&StartAt=0&NbRecs=10) | *Nicotiana tabacum* | ATTTTCTCCA | Cis-acting element involved in defense and stress responsiveness |
| BrPDI11-2 | [ABRE](http://bioinformatics.psb.ugent.be/webtools/plantcare/cgi-bin/show_site_info.htpl?QWhere=ID_of_Site like 'AT~ABRE'&StartAt=0&NbRecs=10) | A. thaliana | TACGTG | Cis-acting element involved in the abscisic acid responsiveness |
|  | Circadian | *S. lycopersicum* | CAANNNNATC | *Cis* acting regulatory element involved in circadian control |
|  | LTR | *A. thaliana* | CCGAAA | Low-temperature-responsive |
|  | [MRE](http://bioinformatics.psb.ugent.be/webtools/plantcare/cgi-bin/show_site_info.htpl?QWhere=ID_of_Site like 'PC~MRE'&StartAt=0&NbRecs=10) | *P. crispum* | AACCTAA | MYB binding site involved in light responsiveness |
|  | TCA-element | *B. oleracea* | GAGAAGAATA | *Cis-*acting element involved in salicylic acid responsiveness |
|  | WBOX | *A. thaliana* | TTGAC | Functions in response to environmental stresses/ regulated plant defense response |
| BrPDI11-3 | Circadian | *S. lycopersicum* | CAANNNNATC | *Cis* acting regulatory element involved in circadian control |
|  | LTR | *A. thaliana* | CCGAAA | Low-temperature-responsive |
|  | [MRE](http://bioinformatics.psb.ugent.be/webtools/plantcare/cgi-bin/show_site_info.htpl?QWhere=ID_of_Site like 'PC~MRE'&StartAt=0&NbRecs=10) | *P. crispum* | AACCTAA | MYB binding site involved in light responsiveness |
|  | TCA-element | *B. oleracea* | GAGAAGAATA | *Cis-*acting element involved in salicylic acid responsiveness |
| BrPDI11-4 | [ABRE](http://bioinformatics.psb.ugent.be/webtools/plantcare/cgi-bin/show_site_info.htpl?QWhere=ID_of_Site like 'AT~ABRE'&StartAt=0&NbRecs=10) | A. thaliana | TACGTG | Cis-acting element involved in the abscisic acid responsiveness |
|  | Circadian | *S. lycopersicum* | CAANNNNATC | *Cis* acting regulatory element involved in circadian control |
|  | HSE | *B. oleracea* | AGAAAATTCG | *Cis*-acting element involved in heat stress responsiveness |
|  | LTR | *A. thaliana* | CCGAAA | Low-temperature-responsive |
|  | [MRE](http://bioinformatics.psb.ugent.be/webtools/plantcare/cgi-bin/show_site_info.htpl?QWhere=ID_of_Site like 'PC~MRE'&StartAt=0&NbRecs=10) | *P. crispum* | AACCTAA | MYB binding site involved in light responsiveness |

**Table S2** A total of 76 *PDI* and *PDIL* genes name and accession numbers from 4 species used for constructing phylogenetic tree, including 32 from *Brassica rapa,*(*Br*) 21 from *Arabidopsis thaliana* (*At*)*,*11 from *Brachypodium distachyon* (*Bd*) and 12 from *Zea mays* (*Zm*)

| **Gene Name** | **Accession number** | **Gene Name** | **Accession number** | **Gene Name** | **Accession number** |
| --- | --- | --- | --- | --- | --- |
| BrPDI1-1 | Bra016405 | AtPDIL1-1 | [AT1G21750](https://www.arabidopsis.org/servlets/TairObject?id=31012&type=locus) | ZmPDIL1-1 | GRMZM2G091481_T01 |
| BrPDI1-2 | Bra012293 | AtPDIL1-2 | AT1G77510 | ZmPDIL1-2 | GRMZM2G163421_T01 |
| BrPDI1-3 | Bra017948 | AtPDIL2-1 | [AT3G54960](https://www.arabidopsis.org/servlets/TairObject?id=37061&type=locus) | ZmPDIL2-1 | GRMZM2G134889_T01 |
| BrPDI1-4 | Bra008311 | AtPDIL2-2 | [AT5G60640](https://www.arabidopsis.org/servlets/TairObject?id=134892&type=locus) | ZmPDIL2-2 | GRMZM2G033829_T01 |
| BrPDI1-5 | Bra015665 | AtPDIL3-1 | [AT1G52260](https://www.arabidopsis.org/servlets/TairObject?id=28354&type=locus) | ZmPDIL3-1 | GRMZM2G014076_T01 |
| BrPDI2-1 | Bra007120 | AtPDIL3-2 | [AT3G16110](https://www.arabidopsis.org/servlets/TairObject?id=38976&type=locus) | ZmPDIL4-1 | GRMZM2G128171_T03 |
| BrPDI2-2 | Bra002464 | AtPDIL4-1 | [AT2G47470](https://www.arabidopsis.org/servlets/TairObject?id=34903&type=locus) | ZmPDIL4-2 | GRMZM2G159369_T01 |
| BrPDI2-3 | Bra020239 | AtPDIL5-1 | [AT1G04980](https://www.arabidopsis.org/servlets/TairObject?type=gene&id=28583) | ZmPDIL5-1 | GRMZM2G389173_T01 |
| BrPDI3-1 | Bra014319 | AtPDIL5-2 | [AT2G32920](https://www.arabidopsis.org/servlets/TairObject?id=34469&type=locus) | ZmPDIL6-1 | GRMZM2G073628_T01 |
| BrPDI3-2 | Bra018958 | AtPDIL6-1 | [AT1G07960](https://www.arabidopsis.org/servlets/TairObject?id=137689&type=locus) | ZmPDIL7-1 | GRMZM2G176443_T02 |
| BrPDI4-1 | Bra000454 | AtPDIL7-1 | [AT1G35620](https://www.arabidopsis.org/servlets/TairObject?id=27913&type=locus) | ZmPDIL7-2 | GRMZM2G007385_T01 |
| BrPDI4-2 | Bra004455 | AtPDIL8-1 | [AT3G20560](https://www.arabidopsis.org/servlets/TairObject?id=37605&type=locus) | ZmPDIL8-1 | GRMZM2G067063_T01 |
| BrPDI5-1 | Bra015375 | AtPDIL8-2 | [AT4G27080](https://www.arabidopsis.org/servlets/TairObject?id=129674&type=locus) |  |  |
| BrPDI5-2 | Bra005546 | AtPDIL8-3 | AT1G50950 |  |  |
| BrPDI6-1 | Bra018672 | AtPDIL9-1 | AT1G15020 |  |  |
| BrPDI7-1 | Bra034408 | AtPDIL9-2 | AT2G01270 |  |  |
| BrPDI8-1 | Bra001793 | AtPDIL10-1 | AT3G03860 |  |  |
| BrPDI8-2 | Bra035770 | AtPDIL10-2 | AT1G34780 |  |  |
| BrPDI8-3 | Bra019071 | AtPDIL11-1 | AT4G21990 |  |  |
| BrPDI8-4 | Bra010413 | AtPDIL11-2 | AT1G62180 |  |  |
| BrPDI8-5 | Bra030465 | AtPDIL11-3 | AT4G04610 |  |  |
| BrPDI8-6 | Bra018881 | BdPDIL1-1 | Bradi4g23180 |  |  |
| BrPDI9-1 | Bra026786 | BdPDIL1-2 | Bradi5g10610 |  |  |
| BrPDI9-2 | Bra014330 | BdPDIL2-1 | Bradi3g00210 |  |  |
| BrPDI10-1 | Bra001092 | BdPDIL3-1 | Bradi1g48460 |  |  |
| BrPDI10-2 | Bra031969 | BdPDIL4-1 | Bradi2g12560 |  |  |
| BrPDI10-3 | Bra036758 | BdPDIL4-2 | Bradi2g35020 |  |  |
| BrPDI10-4 | Bra036429 | BdPDIL5-1 | Bradi4g31830 |  |  |
| BrPDI11-1 | Bra019406 | BdPDIL6-1 | Bradi1g65710 |  |  |
| BrPDI11-2 | Bra013579 | BdPDIL7-1 | Bradi5g10380 |  |  |
| BrPDI11-3 | Bra034466 | BdPDIL7-2 | Bradi3g45540 |  |  |
| BrPDI11-4 | Bra029505 | BdPDIL8-1 | Bradi1g25977 |  |  |
| BrPDI1-1 | Bra016405 |  |  |  |  |

**Table S3** Primers for RT-PCR andreal-time PCR analysis of 32 *BrPDI* genes

| **Gene name** | **Forward** | **Reverse** |
| --- | --- | --- |
| BrPDI1-1 | GAGACCAACAGAGAGTTTGC | CACCAACCACAATAACCTTC |
| BrPDI1-2 | TCTTTGACAGCCCTAACACT | CTCCACATTTGTTTTCAGGT |
| BrPDI1-3 | GATCCTTGTGTTCTCGTTGT | GGACATGACTGCTCAATTCT |
| BrPDI1-4 | GTTGTCGGTGAAAAGAATGT | CCTTGGAATCAACAAACAGT |
| BrPDI1-5 | GCTAGCGAGGAGTCTAACAA | TTCTCACCAATAACCTCACC |
| BrPDI2-1 | TGGCTCAGAAGTACGAGATT | CGAGAAAAGCAAGAACAACT |
| BrPDI2-2 | TTCAACGAAGGAGAAGAAGA | GCGTAGAACTCGACCATAAC |
| BrPDI2-3 | CTTTACACTGGTGGAAGGAC | GGGTTCACTGTTTGGTAGAA |
| BrPDI3-1 | ACAGCTCCTAGCAGTAGACG | GAACTCCCAATCTCTTTCAA |
| BrPDI3-2 | CGAAACTTCTGTTTCCTGAG | GCTGAGCCATATTCTGAAAG |
| BrPDI4-1 | TAGTGGTTATCCCACCATTC | ACGAGGACATCTTTGTTCTG |
| BrPDI4-2 | GAAGGAGGTACCAATGTCAA | CATCCAAGTTGGCTATGACT |
| BrPDI5-1 | TACTGTGGCTGCTATTGATG | ACTAGGCTCGGACTTCTTCT |
| BrPDI5-2 | TGGCTAGTGTTTTGAAAGGT | TCAGAAGAGCCTTAATCTGC |
| BrPDI6-1 | ACTGCGTGGTTTGTAAAGTT | CAACGTCTCTTTTACCTTGG |
| BrPDI7-1 | AGTTTGCAGACTCGTTTCAT | ATCCCATAAAAGATGGTCCT |
| BrPDI8-1 | TGTGAATGACGTGTTAGGAA | ATAACGGAAAGTGGTGTGAC |
| BrPDI8-3 | CCAAGAAGCTGATCTCTGTC | GTCGGATTTGTCCTCTAATG |
| BrPDI8-2 | GTCGCATGTTGTTAGCCA | CATATTCCTCAACCAATG |
| BrPDI8-4 | CACTCAGGAAGCTGATCTCT | GCCTGATTTGTCCTCTAATG |
| BrPDI8-5 | ACGCATTGATTTCAACGTCA | TGACATCAACCGATGCAAAT |
| BrPDI8-6 | CAGGTTCCTGGTGAACTCGT | AAGGCAGTAGCCGCTTCATA |
| BrPDI9-1 | TGGTTCCACATCATCCTTCA | CATGAGGGAGATCCTTTCCA |
| BrPDI9-2 | CGAATTGAACAGCTCCAACT | TCAAAACGATTCCAGGATGT |
| BrPDI10-1 | ACCAGCAACCACCTTAGACG | CAGGTTCAGATTGGGAGCAT |
| BrDPI10-2 | TGTCCAGTACGTGGCAGAAG | AAGACGAGGATTGCCATTTG |
| BrDPI10-3 | ACGTTCCCAATCTGAACCTG | TGAAGTTTGGCCTAGGGAGA |
| BrDPI10-4 | GTTTTCCCTGCACTGGTTGT | TGCTGTTTGATGAGCTGGAC |
| BrDPI11-1 | TGTTTCCTGACGCTGTTGAG | TTTTCTTTGTCCCGTGATCC |
| BrDPI11-2 | TTGAGGTCCAAGCTCTGGTT | CCACCGTCTAACCCTTCAAA |
| BrDPI11-3 | AAGCTTGGTAACCGCAAAGA | CTTGCTTGGCAAACTCCTTC |
| BrDPI11-4 | TTTTGCAGAGCTTGCTGAGA | GAGCAACATCTTCAGCACCA |
| *BrRTActin* | ATGGTTGGGATGGGTCAAAAA | TCTTTAATGTCACGGACGATT |
| Real time | CAACCAATCGTCTGTGACAA | ATGTCTTGGCCTACCAACAA |
